# Optimisation of quantitative brain diffusion-relaxation MRI acquisition protocols with physics-informed machine learning

**DOI:** 10.1016/j.media.2024.103134

**Published:** 2024-03-05

**Authors:** Álvaro Planchuelo-Gómez, Maxime Descoteaux, Hugo Larochelle, Jana Hutter, Derek K. Jones, Chantal M.W. Tax

**Affiliations:** a Cardiff University Brain Research Imaging Centre (CUBRIC), School of Psychology, Cardiff University, Cardiff, United Kingdom; b Imaging Processing Laboratory, Universidad de Valladolid, Valladolid, Spain; c Sherbrooke Connectivity Imaging Lab (SCIL), Computer Science Department, Université de Sherbrooke, Sherbrooke, QC, Canada; d Google DeepMind, Montréal, QC, Canada; e Centre for Medical Engineering, Centre for the Developing Brain, King’s College London, London, United Kingdom; f Image Sciences Institute, University Medical Center Utrecht, Utrecht, The Netherlands; g Cardiff University Brain Research Imaging Centre (CUBRIC), School of Physics and Astronomy, Cardiff University, Cardiff, United Kingdom

**Keywords:** Quantitative MRI, Machine learning, Brain, Diffusion-relaxation

## Abstract

Diffusion-relaxation MRI aims to extract quantitative measures that characterise microstructural tissue properties such as orientation, size, and shape, but long acquisition times are typically required. This work proposes a physics-informed learning framework to extract an optimal subset of diffusion-relaxation MRI measurements for enabling shorter acquisition times, predict non-measured signals, and estimate quantitative parameters.

*In vivo* and synthetic brain 5D-Diffusion-$T_{1}-T_{2}^{*}$-weighted MRI data obtained from five healthy subjects were used for training and validation, and from a sixth participant for testing. One fully data-driven and two physics-informed machine learning methods were implemented and compared to two manual selection procedures and Cramér–Rao lower bound optimisation.

The physics-informed approaches could identify measurement-subsets that yielded more consistently accurate parameter estimates in simulations than other approaches, with similar signal prediction error. Five-fold shorter protocols yielded error distributions of estimated quantitative parameters with very small effect sizes compared to estimates from the full protocol. Selected subsets commonly included a denser sampling of the shortest and longest inversion time, lowest echo time, and high b-value.

The proposed framework combining machine learning and MRI physics offers a promising approach to develop shorter imaging protocols without compromising the quality of parameter estimates and signal predictions.

## 1 Introduction

Clinical magnetic resonance images (MRI) typically show qualitative tissue contrast, with intensities arbitrarily scaled according to different MR-phenomena. Quantitative MRI, on the other hand, aims to extract reproducible measures more directly related to tissue properties that can be studied longitudinally and/or across populations. Examples of the phenomena that can be studied to assess tissue changes quantitatively in health and disease are diffusion and relaxation (Cercignani and Bouyagoub, 2018).

To quantify tissue properties, distinct MRI experiments are typically performed to probe and subsequently quantify each individual phenomenon by acquiring multiple images in which one or a few experimental MRI parameters are varied. Diffusion MRI (dMRI) sensitises the MRI signal to the random molecular motion of water by acquiring MR data with externally applied field gradients with different amplitudes and along different orientations. Relaxation MRI measures the return to equilibrium of nuclear spin polarisation after a radiofrequency pulse, and relaxation times can provide valuable insights into tissue composition and pathology. Notwithstanding the ultimate promise of quantitative diffusion and relaxation measures to improve diagnosis, disease monitoring, and understanding of pathophysiology, accurate and precise quantification – particularly the characterisation of multiple distinct tissue compartments within a voxel – commonly requires acquisition times beyond the realm of clinical adoption.

Moreover, each individual MRI contrast provides only part of the picture. In brain white matter (WM), for example, dMRI can disentangleintra- and extra-axonal water motion but is only minimally sensitive to water trapped between the insulating myelin layers, whereas relaxation MRI, particularly at short echo time, has sensitivity to myelin water but can typically not separate intra- and extra-axonal water. As such, rather than acquiring diffusion and relaxation contrasts separately, recent works have adopted the simultaneous variation of multiple experimental variables to optimally exploit their joint and complementary information (de Almeida Martins et al., 2021b; Slator et al., 2021; Tax, 2020) inspired by approaches common in physical chemistry NMR (Lindblom et al., 1977). Correlation experiments offer valuable insights, but the soaring dimensionality of MRI acquisition parameters makes diffusion-relaxation MRI designs frequently ad hoc and time-inefficient, particularly when resolving orientational features in dMRI. Faster image readout strategies, e.g. through compressed sensing, provide exciting opportunities to acquire more images per unit time (Lustig et al., 2007). Still, the question remains which parts of the high-dimensional parameter space contain the most important information, and which trade-offs need to be made to sample the extremities of the space while adhering to hardware constraints.

Experimental design to optimise the MRI acquisition has been an active area of research, with most approaches taking a ‘top-down’ or *model-based* strategy by assuming that it is known which tissue properties/physical phenomena are important to quantify. Alternatively, a ‘bottom-up’ *data-driven* strategy can identify measurements containing the most important information without any prior assumptions about the relevant parameters to be quantified or their distribution.

The top-down optimisation methods commonly rely on *a priori* analytical expressions that link the measured signals to relevant parameters such as compartmental diffusivities, relaxation times, and signal fractions. Optimisation approaches include minimisation of parameter variance through optimisation of the Cramér–Rao lower bound (CRLB) or features of the Fisher information matrix (Alexander, 2008; Coelho et al., 2019; Lampinen et al., 2020) and Monte Carlo simulations (Lemke et al., 2011). Knutsson (2019) simulated distributions of tissue compartments and optimised dMRI protocols based on the mutual information between measurements. However, several tissue properties such as compartmental-exchange times or fibre undulation often remain elusive because analytical models become intractable.

*Data-driven* methods can use Machine Learning (ML) to establish complex non-linear relationships between signals and parameters. Two data-driven methods were implemented in the MICCAI MUlti-DIffusion (MUDI) challenge (Pizzolato et al., 2020) aiming at selecting an optimal subsets of MRI volumes to predict the whole set of MR signals. The sub-protocols resulting from these methods could achieve better predictions than other model-based approaches. The *Select and retrieve via direct upsampling network* (SARDU-Net) approach (Grussu et al., 2021b) concatenates two fully-connected neural networks (NN), where the first network is used to extract the optimal subset of MRI measurements with the highest weights from a comprehensively-sampled dataset, and the second to predict the MR signal of the whole dataset from the reduced dataset. In the MUDI challenge, a method based on the use of concrete autoencoders, a neural network which extracts an optimal subset of measurements (Abid et al., 2019; Maddison et al., 2016; Tax et al., 2021b) consistently showed the lowest prediction error when reducing the number of selected measurements. However, the design of these methods for optimising protocols to accurately predict the MRI signal does not implicitly consider the importance of accurately estimating quantitative parameters.

In this work, we propose data-driven and physics-informed bottom-up approaches to reconstruct the MR signal and characterise diffusion and relaxation phenomena with a reduced number of MRI measurements. Starting with rich multi-dimensional data, the approach seeks the most important signals to acquire, which can be cast as a feature selection (i.e., measurement-subset selection) problem. We build on the concrete autoencoder approach described in Tax et al. (2021b) to develop a physics-informed data-driven methods that select the most representative measurements of an MRI dataset guided by a signal representation.

Our hypothesis is that physics-informed optimisation identifies subsets of MRI measurements that provide, per unit time, better estimates of quantitative parameters characterising diffusion and relaxation compared to full data-driven or other methods. To test this hypothesis, the estimation of quantitative parameters and prediction of the MRI signal was compared between the proposed approach, the original concrete autoencoder method, manually selected protocols, and CRLB optimisation.

## 2 Materials and methods

### 2.1 Data

#### 2.1.1 In vivo data

The main dataset used for training and validation was composed of five controls (age between 19 and 46 years, three women) who were scanned after informed consent was obtained (West London REC 12/LO/1247) on a 3T 80 mT/m scanner with a 5D Diffusion-$T_{1}-T_{2}^{*}$ protocol varying b-value (trace of the b-tensor, *b*; Westin et al. (2016))^2^, gradient orientation (first eigenvector of the b-tensor (*Θ*_*g*_, *Φ*_*g*_)), inversion time TI and delay time TD (delayed readout with respect to the echo using an asymmetric spin echo) (Pizzolato et al., 2020; Hutter et al., 2018) (1344 unique settings). Specifically, b-values included [0, 500, 1000, 2000, 3000] s/mm^2^, TD [0, 25, 50] ms and TI ranged between 20 and 7322.7 ms. Repetition time TR and b-tensor anisotropy *b*_*Δ*_ were 7500 ms and 1, respectively. In addition to the main dataset, data from another healthy subject with the same acquisition parameters were employed for testing.

The data were preprocessed as previously described (Pizzolato et al., 2020). Briefly, this included denoising in the complex domain based on the Marchenko–Pastur Principal Component Analysis (MP-PCA) procedure (Cordero-Grande et al., 2019), co-registration of the diffusion-weighted images using Dipy (Garyfallidis et al., 2014), and correction for susceptibility-induced distortion (Andersson et al., 2003; Smith et al., 2004).

A global scaling factor between each subject and the first subject was estimated from the median of the images, and intensities were subsequently divided by the 95th-percentile intensity of subject 1.

White matter (WM), grey matter (GM) and cerebrospinal fluid (CSF) masks were obtained through multi-tissue constrained spherical deconvolution (Jeurissen et al., 2014; Dhollander et al., 2019), and voxels classified as WM, GM and CSF to enable evaluation per tissue type.

#### 2.1.2 Synthetic data

Synthetic signals were generated based on the *in vivo* data to evaluate the accuracy of the estimated parameter maps from subsets of measurements. The use of synthetic data provided a ground truth for the maps and provided a mean to evaluate accuracy, which is not possible for the *in vivo* data. The following signal equation was fitted to the *in vivo* data using a NN as described in Section 2.4 using all the acquired measurements (Hutter et al., 2018; Tax et al., 2021a):

(1) $$S=S_{0}e^{-b:D}\left|1-2e^{-\frac{\text{TI}}{\text{T}1}}+e^{-\frac{\text{TR}}{\text{T}1}}\right|e^{-\frac{\text{TD}}{\text{T}2^{*}}},$$

where:

(2) $$\begin{array}{l} b:D=(\frac{1}{3}bb_{\Delta }\left[D_{\|}-D_{⊥}\right]-\frac{1}{3}b\left[D_{⊥}+2D_{\|}\right]\\ \;-bb_{\Delta }{\left[\left(\Theta_{g},\Phi_{g}\right)\cdot (\theta ,\phi )\right]}^{2}\left[D_{\|}-D_{⊥}\right]). \end{array}$$

Hence, the ‘ground truth’ maps reflect a realistic distribution of tissue parameters as can be found *in vivo* data. The parameter-ranges were as follows: *S*_0_ [0.5–5], longitudinal relaxation time *T*_1_[100–5000 ms], transverse relaxation time $T_{2}^{*}$ [0.01–2000 ms], parallel diffusivity *D*_∥_ [0.01–3.2 μm^2^/ms], perpendicular diffusivity *D*_⊥_ implemented as *k* ⋅ *D*_∥_ [*k* = 0.01–1], and first eigenvector angles *θ* [0-*π*] and *ϕ* [0-(2*π*−0.001)].

Next, considering the estimated parameters on the full dataset as the ground truth, noiseless signals were computed by applying Eq. (1). Finally, Gaussian noise was added with a signal-to-noise ratio (SNR) of 30 for minimum *b*-value (0), TD (0) and maximum TI (7322.7 ms).

### 2.2 Sub-selection of measurements

**Fig. 1:**
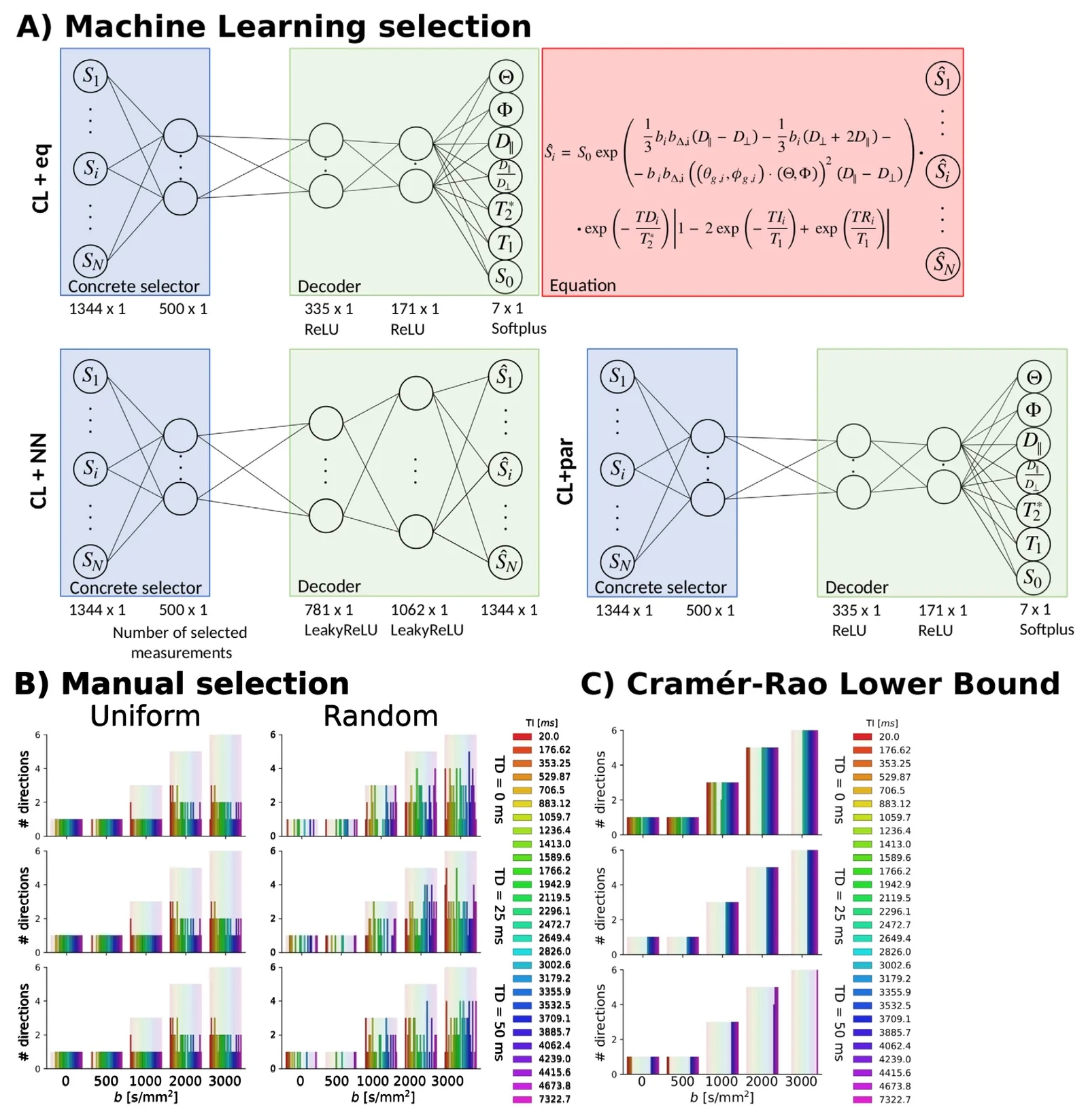
An overview of the methods used to select a subset of MRI measurements. (A) Networks with a concrete selector layer (CL). CL+eq simultaneously selects a subset of volumes and estimates the parameters based on Eqs. (1) and (2). CL+NN is a pure data-driven technique with a fully-connected decoder. CL+par has as output directly the estimated parameters (based on the full dataset) instead of the signal. (B) Measurements selected for the two manual selection procedures. For each method, Uniform or Random, examples of subsets with 500 are shown. (C) Example with the 500 measurements selected for the Cramér–Rao Lower Bound-based procedure. The translucent histograms represent the original set of MRI volumes.

Three ML-based selection procedures were implemented (one fully data-driven and two physics-informed, Fig. 1A), as well as two manual selection strategies: uniform reduction of gradient directions (Uniform), and random selection of volumes (Random), examples in Fig. 1B. Furthermore, a comparison was performed with discrete selection of measurements based on a continuous optimisation of the CRLB (Alexander, 2008). The selected subprotocols were composed of *N* measurements equal to 500, 250, 100 and 50 measurements out of a total of 1344.

#### 2.2.1 Selection layer

All ML-based selections of a subset of *N* MRI volumes were based on a concrete autoencoder (Abid et al., 2019; Maddison et al., 2016; Tax et al., 2021b) with an additional regularisation procedure to avoid the selection of repeated measurements (Strypsteen and Bertrand, 2021). Concrete autoencoders have shown to predict non-selected measurements from a subset of measurements with higher accuracy than other techniques (Abid et al., 2019; Pizzolato et al., 2020).

Specifically, the encoder was a concrete selection layer (CL) that selected the optimal subset of measurements through stochastic linear combinations of input features (Abid et al., 2019). These combinations were controlled by a temperature parameter that gradually decreased its value throughout training. The temperature controls the continuous relaxation of a one-hot vector that determines the sampling of a concrete random variable, i.e., the selection of the optimal subset of measurements. With high temperature values, the output of the CL is a linear combination of features. Low temperature values restrict the number of linear combinations of features in the output of the CL, promoting the extraction of the most informative individual measurements. Two regularisation parameters (Strypsteen and Bertrand, 2021) were employed. The first was a threshold that applied a penalisation when the sum of the probabilities across the selection neurons exceeded the threshold value. Lower values of the threshold were associated with higher penalisation of the selection of duplicated measurements, restricting the potential combinations for the selection. The second parameter determined the strength of the regularisation, where high values promote the selection of non-duplicate measurements at the cost of reducing the weight of data loss (mean-squared error; MSE) term and potentially increasing the loss.

To train the CL, the maximum temperature was set to 10, and the minimum to 0.1 as in Abid et al. (2019), Tax et al. (2021b). For the regularisation, the threshold value was set to 1, and the strength parameter was set to 0.1 similar to Strypsteen and Bertrand (2021), which achieved the selection of non-duplicate measurements for each *N*.

#### 2.2.2 Decoder

The decoder for signal prediction was either purely data-driven (one method) or physics-informed (two methods). These methods are represented graphically in Fig. 1A. Furthermore, the decoder either aimed to predict the MRI signal (loss computed with respect to the measured signal) or quantitative parameters (loss computed with respect to estimated parameters on all measurements).

The first ML technique with loss based on the MRI signal was CL+eq (concrete layer encoder and decoder with signal equation). This decoder was a physics-informed technique that combined a NN with a physical signal equation. The network was composed of two hidden layers, whose number of nodes was determined with a one dimensional linear interpolation procedure between the number of selected measurements and the number of parameters (7), ReLU as activation function, and a final Softplus layer. The first layer of the decoder was composed of seven nodes corresponding to the parameters in Eq. (1). Thus, this method simultaneously sub-selects measurements, estimates the physical parameters, and predicts the signal.

The full data-driven ML method with loss based on the MRI signal, coined CL+NN (concrete layer encoder and neural network decoder), consisted of a decoder with two hidden fully-connected layers of variable number of nodes depending on the number of selected measurements, and Leaky Rectified Linear Units (ReLU) as activation functions. The number of nodes for the two layers was extracted through one dimensional linear interpolation between the number of selected measurements and the total number of MRI measurements (1344).

The ML physics-informed method with loss based on the quantitative parameters, CL+par (concrete layer encoder and decoder to directly predict the parameters), was a fully-connected NN that used the parameters estimated on the full dataset (i.e. the ground truth parameters in synthetic experiments evaluating the best-case scenario) to compute the loss instead of the MRI signal as in CL+eq. Therefore, Eqs. (1) and (2) were only applied before training. To avoid bias during training caused by the different ranges of each parameter, the parameters were normalised as:

(3) $${\hat{x}}_{i}=\frac{x_{i}-x_{i,\min}}{x_{i,\max}-x_{i,\min}},$$

where *x*_*i*_ represents the value for the parameter *i*, one of the seven parameters following Eqs. (1) and (2), and *x*_*i*,min_ and *x*_*i*,max_ are the minimum and the 97.5th percentile of the maximum values of the range for the parameter *i*, described in Section 2.1.2. CL+par was only evaluated in the synthetic experiments where the ground truth maps were available, reflecting the best-case scenario.

#### 2.2.3 Selection based on the CRLB

To compare the selected measurements from ML-based approaches with techniques previously employed to obtain optimal diffusion MRI acquisition parameters, we additionally extracted the optimal sets of measurements following an optimisation of the Fisher information matrix and the CRLB. Specifically, we employed the technique developed by Alexander (2008), but optimising the acquisition parameters relevant to the signal representation in Eqs. (1) and (2), i.e., b-value, TI and TD. The set of gradient directions was fixed as in Alexander (2008) with ‘shells’ of three directions or less to arrive at the desired *N* (Caruyer et al., 2013). It is worth noting that this approach did not subsample a set of prespecified measurements, but directly provided an optimal combination of the parameters for a specific number of measurements. Thus, to select the measurements, those with the lowest overall Euclidean distance on b, TI, and TD (with the parameters normalised to the same order of magnitude and the Hungarian algorithm used for assignment) compared to the set provided by the CRLB-based optimisation were selected (Kuhn, 1955). The average ground truth values in WM and GM of the training subjects were taken as the priors for CRLB optimisation, i.e., *θ* = 1.56 rad, *ϕ* = 1.88 rad, *D*_∥_ = 0.81 μm^2^/ms, *D*_⊥_ = 0.58 μm^2^/ms$T_{2}^{*}=73\text{ms}$, *T*_1_ = 1159 ms, and *S*_0_ = 2.37. This approach was exclusively evaluated on synthetic data where such ground truth values were available.

### 2.3 Network training

Selection and estimation networks were trained based on leave-one-out cross-validation over all subjects from the main dataset (five subjects) using the Adam optimiser (Kingma and Ba, 2014) with learning rate 0.001, batch size 256, and MSE loss. Specifically, the employed loss function was the squared L2 norm, with regularisation as described in Section 2.2.1 for selection networks, and this approach was applied for all the methods. To assess the reproducibility, the sub-selection was repeated with three different seeds. The variability across seeds was compared to the variability of leave-one-out runs across subjects with a single seed, by comparing the standard deviations for each setting of TI, TD, and *b* between the two scenarios.

The dataset used for training comprised data from four subjects, (approximately 80% of available voxels). 20% of voxels (from the remaining subject) were used for validation. All the selection networks with a CL were trained until the mean of the concrete samples was equal or higher than 0.998 or after running 5000 epochs. The mean of the concrete samples is the mean of the maximum value per vector of weights, where a value of 0.998 indicates a close approximation to a one-hot vector.

For the estimation network, the results with the lowest validation error across seeds were saved after running 100 epochs, or 200 epochs in the case of a small number of selected measurements, setting as stopping criterion a validation error lower than 0.00001. To compare the results between estimations from different selection methods, the estimated results from the test subject, not included in any training or validation subset, were computed.

### 2.4 Evaluation of sub-selections

The sub-selections from the ML and manual approaches were evaluated based on the ability to (1) predict all measurements, including the non-selected measurements, and (2) estimate quantitative maps. To this end, a separate network for each of the subsets was trained with a similar structure to the decoder of the CL+eq approach (Fig. 1A), providing predictions of the signals as well as estimated maps. Subsequently, for the unseen test dataset, the error between (1) the predicted and measured signals (noisy signals for the synthetic simulations) and (2) the estimated and ground truth parameter maps (i.e. the parameter maps estimated on all the *in vivo* measurements, see Section 2.1.2) was computed. For the first eigenvector, the error was computed from the dot product of the estimated and ground truth directions. All the errors were obtained in the whole brain, GM and WM, using the masks described in Section 2.1.1. Error distributions over all voxels were investigated. For the predicted signals, distributions of the mean absolute error (MAE) across volumes and their median values were computed, and for the estimated parameters distributions of absolute errors (AE) and their medians were extracted. In addition to errors, the interquartile range (IQR) and coefficient of variation (CV) of errors across all voxels were computed as measures of variability.

To quantify the difference between the errors of the estimations from the selected sub-protocols and those obtained with the whole dataset, the effect size was computed using the Cohen’s d value for paired data as follows:

(4) $$d=\frac{{\bar{x}}_{1}-{\bar{x}}_{2}}{\sigma_{x_{1}-x_{2}}},$$

where ${\bar{x}}_{1,2}$ represents the mean error for each estimation method, and $\sigma_{x1-x2}$ the standard deviation of the difference between the errors for each method. Effect sizes were categorised as very small (0.01 ≤ d < 0.2), small (0.2 ≤ d < 0.5), medium (0.5 ≤ d < 0.8), large (0.8 ≤ d < 1.2), very large (1.2 ≤ d < 2.0) and huge (d ≥ 2.0) (Sawilowsky, 2009).

The effect of the sub-selection on the quantitative maps depends on the estimator, and the described NN-based strategy allowed leveraging the information of all the measurements from the training subjects. This was hypothesised to become more beneficial as the number of selected measurements decreased. To further investigate the effect of estimator, a non-linear least squares (NLS) trust-region-reflective approach – which only obtains estimations from the sub-selected measurements – was also employed.

## 5 Results

### 3.1 Synthetic data

#### 3.1.1 Sub-selection of measurements

**Fig. 2:**
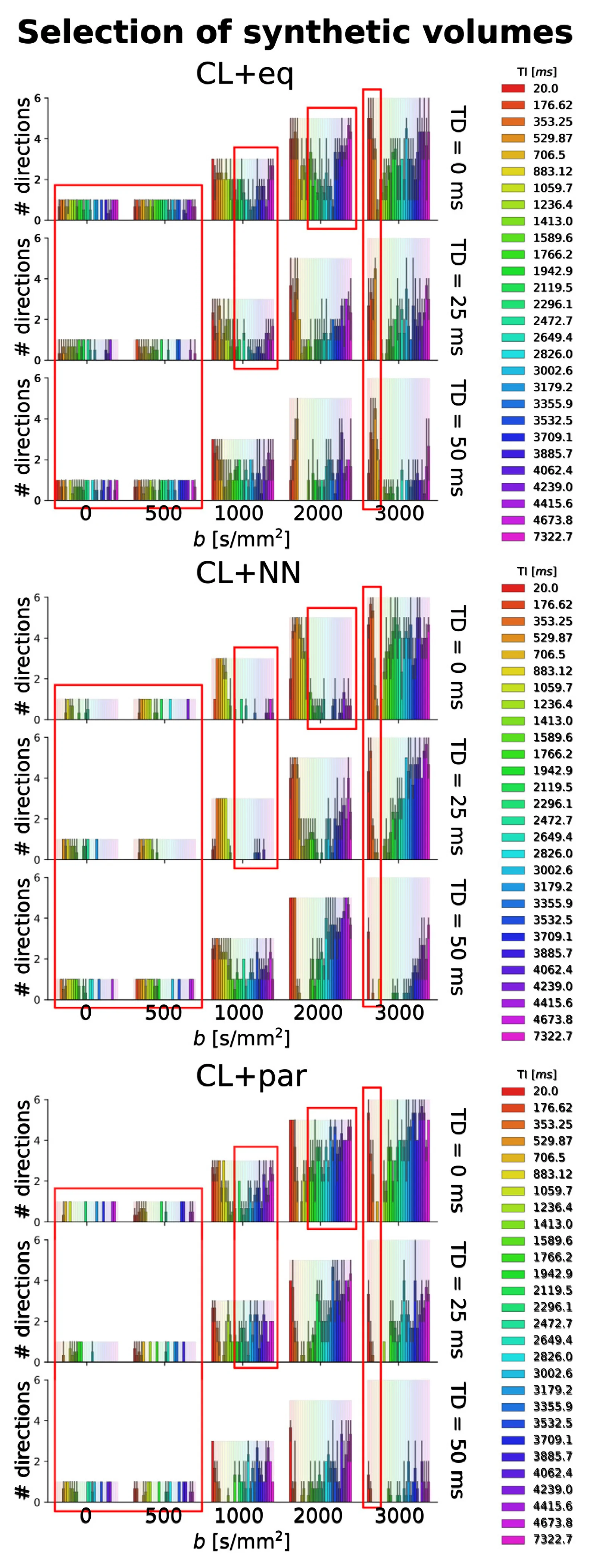
Comparison of the selected 500 measurements between the machine learning methods. The histograms represent the mean number of chosen volumes (gradient orientations) for each b-value (0, 500, 1000, 2000 and 3000 s/mm^2^), TD (0, 25 and 50 ms), and TI (28 values from 20 to 7322.7 ms) across three different seeds. The errorbars represent the standard deviation. The red rectangles highlight the measurements more frequently selected with the physics-informed selection (CL+eq) compared to the pure data-driven selection (CL+NN). The translucent histograms show the original distribution.

Fig. 2 shows a comparison of the sub-selection of *N* = 500 measurements across the different ML methods. In this figure, each selection method was trained three times with different initialisation seeds on the same training and validation subjects (validation subject 3, training carried out with the other subjects).

All methods consistently selected low and high TI for higher b-values (1000, 2000 and 3000 s/mm^2^), and more measurements for the lowest TD. CL+eq more frequently selected lower b-values (0 and 500 s/mm^2^) than the other methods, together with the lowest TI values for all b-values. CL+NN selected fewer volumes with medium-high TI for all b-values except 3000 s/mm^2^. CL+par selected similar volumes compared to CL+eq, albeit fewer medium-low TI values for all b-values, particularly at b = 3000 s/mm^2^ and higher TD. Results were similar when assessing the variability in selection across different subjects and one fixed seed, as shown in Supplementary Figure 1.

Regarding the variability of the selection across seeds (single subject) and across subjects (single seed), the average standard deviation for each setting of TI, TD, and *b* was lower across seeds than across subjects for CL+eq (0.517 vs. 0.523) and CL+par (0.470 vs. 0.506), but higher for CL+NN (0.452 vs. 0.402).

The pattern of selections for different numbers of measurements *N* and for the different ML approaches is summarised in Table 1, and the distributions of selected parameters are shown in Supplementary Figure 2. The selection from the Uniform and Random approaches is shown in Fig. 1B for *N* = 500 and in Supplementary Figure 3 for all *N*. Similar patterns were observed for CL+eq and CL+NN, whereas CL+par revealed distinct patterns.

Regarding the CRLB-based selection, the estimated priors were: *θ* = 1.56 rad, *ϕ* = 1.88 rad, *D*_∥_ = 0.81 μm^2^/ms, *D*_⊥_ = 0.58 μm^2^/ms, $T_{2}^{*}$, *T*_1_ = 1159 ms, and *S*_0_ = 2.37. The selections from the CRLB-based optimisations are shown in Fig. 1C for *N* = 500 and in Supplementary Figure 4 for all *N*. Overall, the highest TIs and lowest TDs and b-values were prioritised. The CRLB approach extracted optimal settings to improve the precision per contrast in Eq. (1); for example, for the longest TI and shortest TD, the signal becomes mostly diffusion-weighted and it is exactly these measurements that were frequently selected across the whole range of b-values.

#### 3.1.2 Prediction of signals from a selected subset

**Fig. 3:**
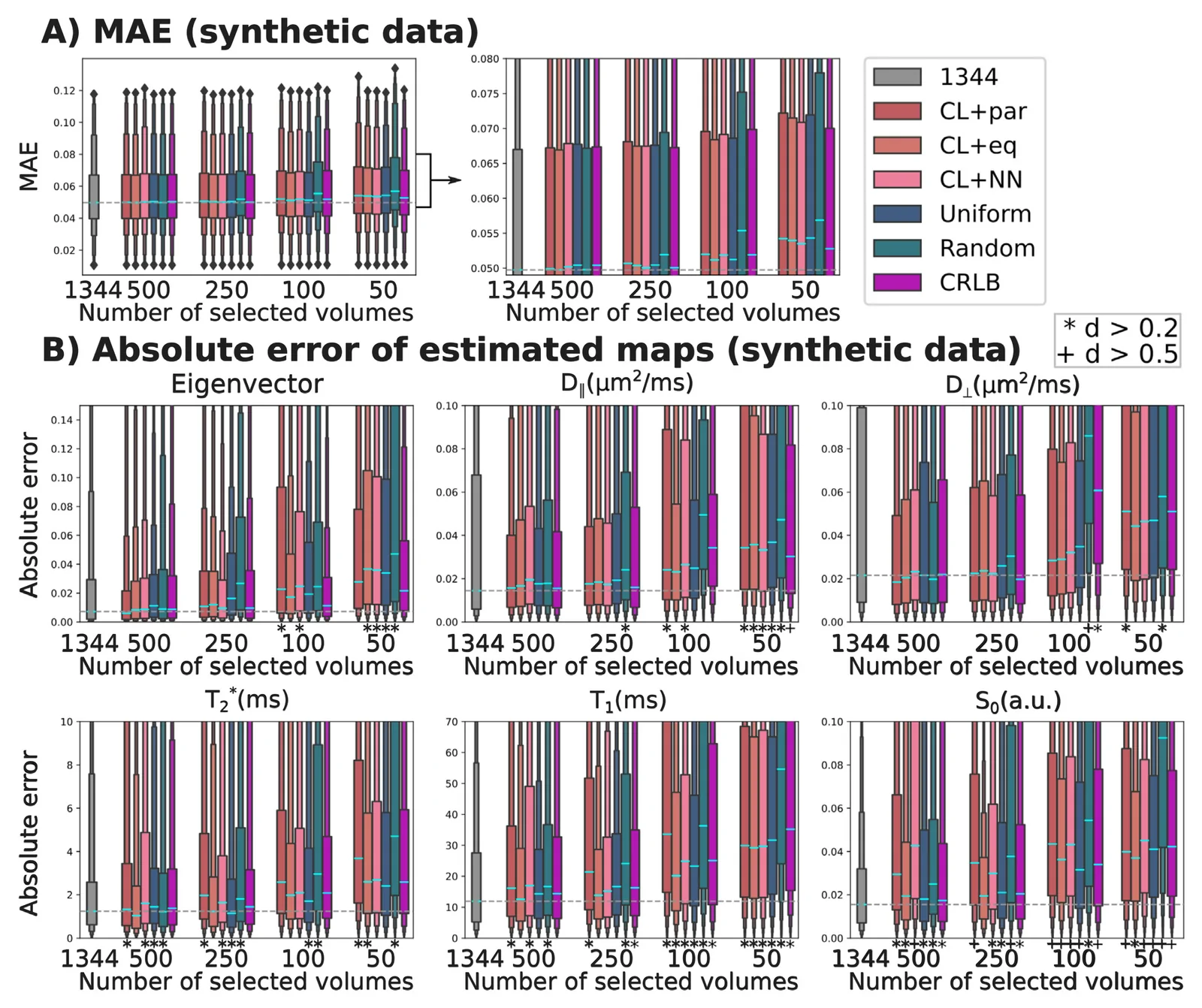
Comparison of the error of the predicted signal (A) and the estimated maps (B) from the synthetic test subject in one of the cross-validation procedures for six selection strategies according to the number of selected measurements. All the error values are shown for the whole brain. In (A), the left subfigure represents the whole range of mean absolute error (MAE) values, and the right subfigure is zoomed between 0.049 and 0.08. For the first eigenvector, the error was represented as one minus the dot product between the estimated and the ground truth maps to facilitate the interpretation. The boxplot 1344 represents the prediction of the whole dataset following the same estimation method as described for the subselected measurements, i.e., the difference between the ground truth and the fitting performed using all the 1344 measurements. The dashed line shows the median error of the 1344 estimation. The highest errors have been omitted for visual purpose. A.u. = arbitrary units. *;+ = small/medium effect size (d > 0.2; 0.5) of the difference of the errors of the estimated parameters between the estimation from the selected subprotocol and the estimation with the whole dataset.

The MAE across selection procedures is summarised in Fig. 3A and Table 2. The “best case” scenario of predicting all measurements from all 1344 noisy synthetic measurements is also shown in Fig. 3A.

ML-based selection methods, particularly CL+eq and CL+NN, resulted in the lowest error for *N* > 50 and the CRLB-based selection presented the lowest MAE for *N* = 50, whereas the Random selection resulted in larger MAE for *N* < 500. Across selection subjects and for *N* = 500, MAE values were similar and CL+eq presented the smallest maximum median among the five leave-one-out runs (Supplementary Table 1). Separating out the prediction of the selected and non-selected measurements (Supplementary Figure 5 A and Supplementary Table 2), CL+eq yielded slightly lower values for the error (all volumes, non-selected volumes, GM and WM). CV/IQR values were lower for CL+eq situations than for CL+NN, but values were similar compared to the other selection approaches. The errors and IQR were higher in GM than in WM, but the CV in WM was higher compared to GM (Supplementary Tables 2–3 and Supplementary Figure 5B).

#### 3.1.3 Parameter estimation

Fig. 3B and Table 2 show errors of quantitative estimates compared to the ground truth; again the “best case” scenario of estimating parameters on all 1344 noisy synthetic measurements is also shown.

For all *N* and quantitative parameters, CL+eq consistently resulted in low median AE, and most frequently performed among the two best methods (14 out of 24 comparisons, i.e. all *N* and all parameters) followed by CRLB (12 out of 24 comparisons), while CL+eq was only once among the two highest median absolute error (eigenvector for *N* = 50). In comparison with the CRLB-based optimisation, CL+eq showed lower errors for $T_{2}^{*}$ (except for *N* = 50) and *T*_1_, but higher errors for *D*_∥_ (except for *N*= 100) and the eigenvector (except for *N* = 500), without clear patterns for *D*_⊥_ and *S*_0_. The effect sizes compared to the 1344 scenario are indicated with symbols (Fig. 3B), revealing consistently very small effect size for CL+eq at *N* = 500 (*d* < 0.2), except for *S*_0_ (*d* < 0.5). Furthermore, CL+eq showed the most consistent very small effect size for all values of *N* (17 out of 24 comparisons), followed by CRLB and Uniform selection (15 and 14 out of 24 comparisons, respectively).

Looking at *N* = 500 across different folds of training and validation subjects, physics-informed networks consistently were amongst the two lowest AE compared to manual approaches (CL+eq in 21 and CL+par also in 21 out of 30 comparisons, Supplementary Figure 6 and Supplementary Table 1). CL+par estimations had the lowest AE for the parameters related to diffusion, i.e., the eigenvector, *D*_∥_ and *D*_⊥_ (11 out of 15 comparisons), *T*_1_ (3 out of 5 comparisons), and *S*_0_ (3 out of 5 comparisons).

**Fig. 4:**
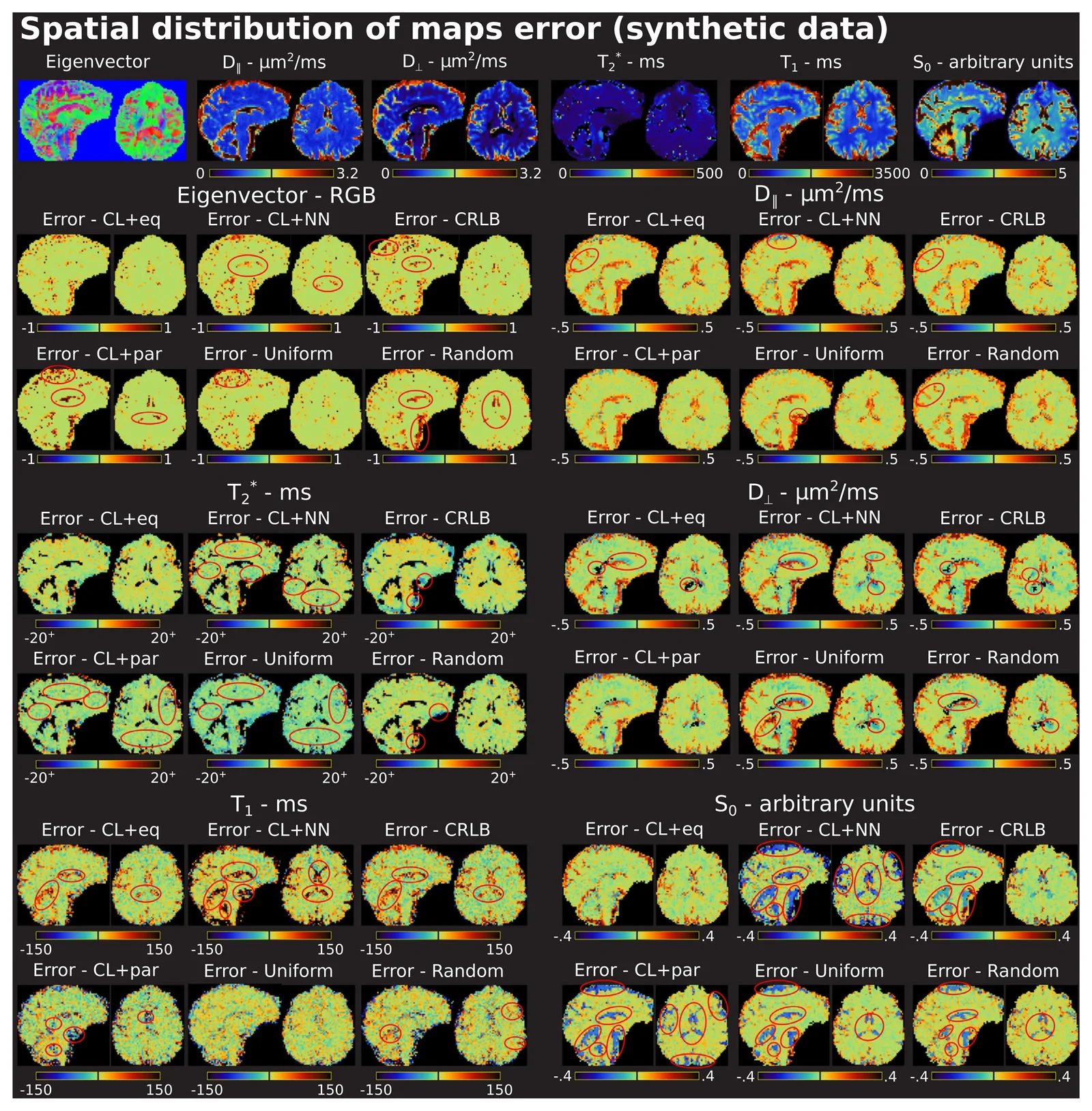
Voxelwise error of the estimated maps from the synthetic test subject for six selection strategies in one of the cross-validation procedures for 500 selected measurements. The first row represents the ground truth for all parameters, and the remaining rows the error considering positive and negative values. The black regions within the brain mask represent values of the errors or parameters outside the shown range. The eigenvector error was represented as one minus the dot product between the estimated and ground truth maps. The black line in the colourbar represents the intermediate value, i.e., 0.5 for the eigenvector error and 0 for the remaining error values (the same for the ground truth bars). The red ellipses remark areas with differences compared to the estimation from the physics-informed selection (CL+eq and/or CL+par), or the Uniform selection for *T*_1_ as it was the method with lower number of areas with higher error. RGB = Red Green Blue. +: The maximum absolute value of the error was considerably higher than 20, but the error was saturated at 20 to better appreciate the errors in the rest of the brain.

In some cases subsampling with *N* = 500 resulted in lower MAE and variability than the best case scenario of all 1344 measurements, e.g. *D*_∥_ and *D*_⊥_; upon closer inspection per tissue type it could be observed that this was primarily due to higher errors in CSF in the latter case (results not shown). Yet, this did not result in notable effect sizes. When assessing GM and WM separately (Supplementary Figure 7 and Supplementary Table 3), the observed patterns were similar to the analysis of the whole brain. Example maps of the spatial distribution of the error values are shown in Fig. 4, and the maps for the different values of *N* are shown in Supplementary Figure 8. Errors were mostly distributed around zero, yet biases were visible in some cases (e.g. negative bias for $T_{2}^{*}$ Uniform subsampling). Regions of high errors showed similar patterns across methods (e.g. CSF).

Considering the variability across voxels, the pattern of methods showing the lowest variability was less obvious. Supplementary Table 4 reports the CV for the test subject averaged across the cross-validation runs, where CL+eq showed lower CV for *T*_1_, the Uniform selection for the first eigenvector, the Random selection for *D*_∥_, *D*_⊥_ and *S*_0_, and CL+NN for $T_{2}^{*}$. Supplementary Table 5 shows the CV and IQR values for different *N*. CL+eq or CL+par presented one of the two lowest IQR across all values of *N*, with two exceptions (*D*_∥_ for *N* = 50, and *S*_0_ for *N* = 500), with the Uniform selection frequently being the other method among the two best cases. For the CV, there was no clear pattern for the lowest values. Additionally, it is worth noting that, where as *N* = 250 resulted in similar median absolute error compared to *N* = 500, the error distribution was wider in general.

Finally, a comparison of quantitative maps from the ML-based estimator and NLS for each number of sub-selected measurements is shown in Supplementary Figure 9 (CL+eq selection is taken as an example). In summary, across all parameters and *N* (500, 250, 100 and 50), the NLS estimation generally showed lower error (68 out of 72 comparisons) and variability (61 out of 72 comparisons). These differences were reduced or inverted for the eigenvector for the lowest number of measurements (100 and 50), except for the WM, where the NLS estimation showed better results. In addition, comparing the NLS estimation for all selection approaches and *N*, the physics-informed networks also showed lower errors compared to the other selection procedures, with the differences between methods being larger compared to the ML estimations (Supplementary Figure 10). CL+eq again showed no effect sizes *d* > 0.2 for *N* ≥ 250, where other methods showed larger effect sizes.

### 3.2 In vivo data

#### 3.2.1 Sub-selection of measurements

**Fig. 5:**
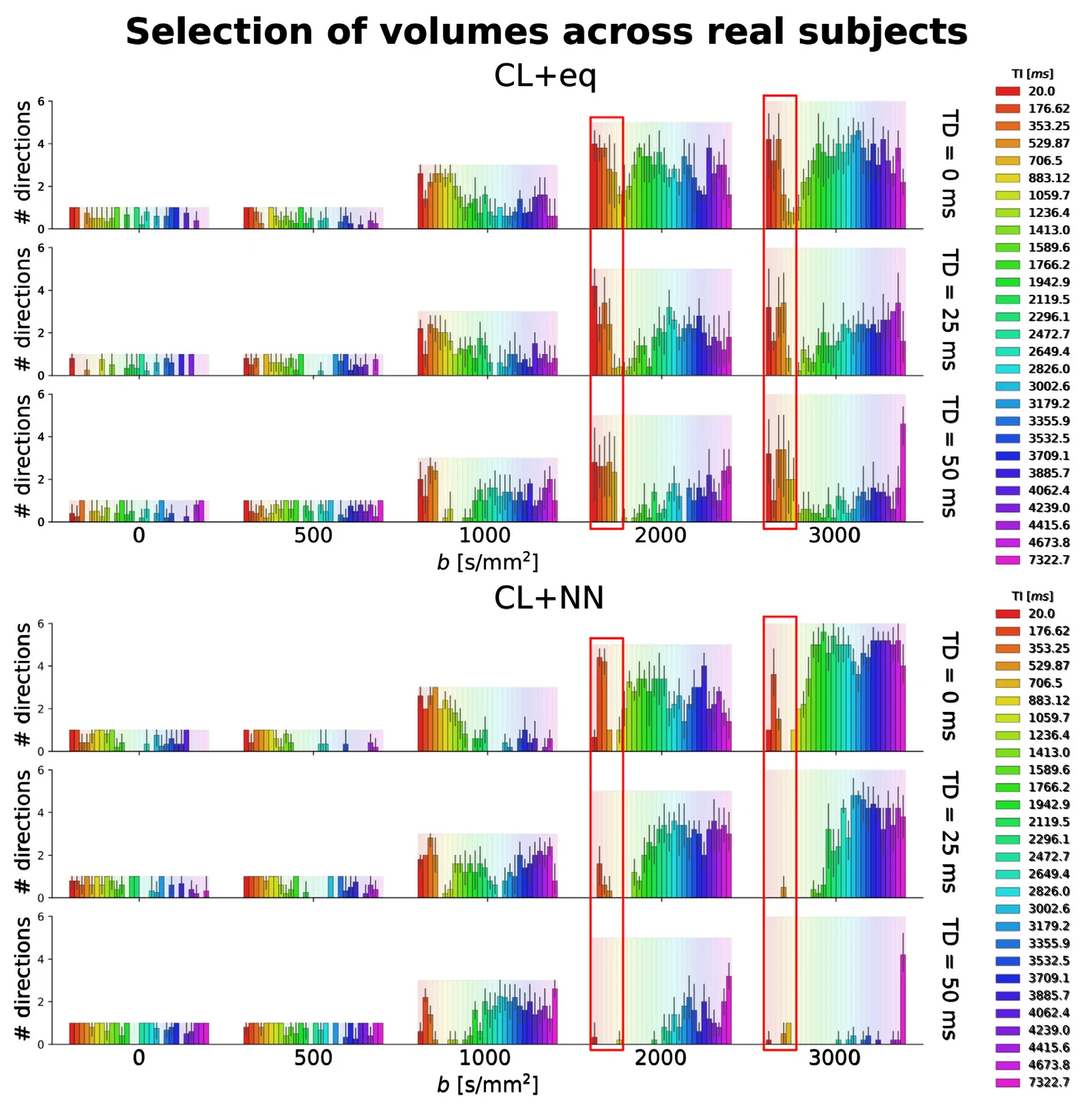
Comparison of the selected 500 measurements between the machine learning methods in *in vivo* data. The histograms represent the mean number of chosen volumes (gradient orientations) for each b-value (0, 500, 1000, 2000 and 3000 s/mm^2^), TD (0, 25 and 50 ms), and TI (28 values from 20 to 7322.7 ms) across five different validation subjects. The errorbars represent the standard deviation. The red rectangles remark the measurements more frequently selected with the physics-informed selection (CL+eq) compared to the pure data-driven selection (CL+NN). The translucent histograms show the original distribution.

The distributions of 500 subselected measurements across validation subjects are shown in Fig. 5. The CL+eq selection resulted in a similar distribution in synthetic and *in vivo* experiments. In contrast, the CL+NN selection showed more pronounced differences between *in vivo* and synthetic data with different sampling patterns for the low TI values between the lowest and highest b-values.

Table 3 summarises the selections when the number of measurements was reduced, and their distributions are shown in Supplementary Figure 11. The patterns of subselection were similar for CL+eq and CL+NN. When reducing the number of selected measurements to 250, CL+eq and CL+NN showed an approximate uniform reduction from the 500 selected measurements. From 250 to 100 measurements, volumes with high b-values and medium TI values were selected with lower frequency for both selection procedures. From 100 to 50 measurements, the reduction of volumes for CL+eq was almost uniformly distributed, and CL+NN presented a lower frequency of volumes with low or medium TI values and non-zero b-values.

#### 3.2.2 Prediction of signals from a selected subset

**Fig. 6:**
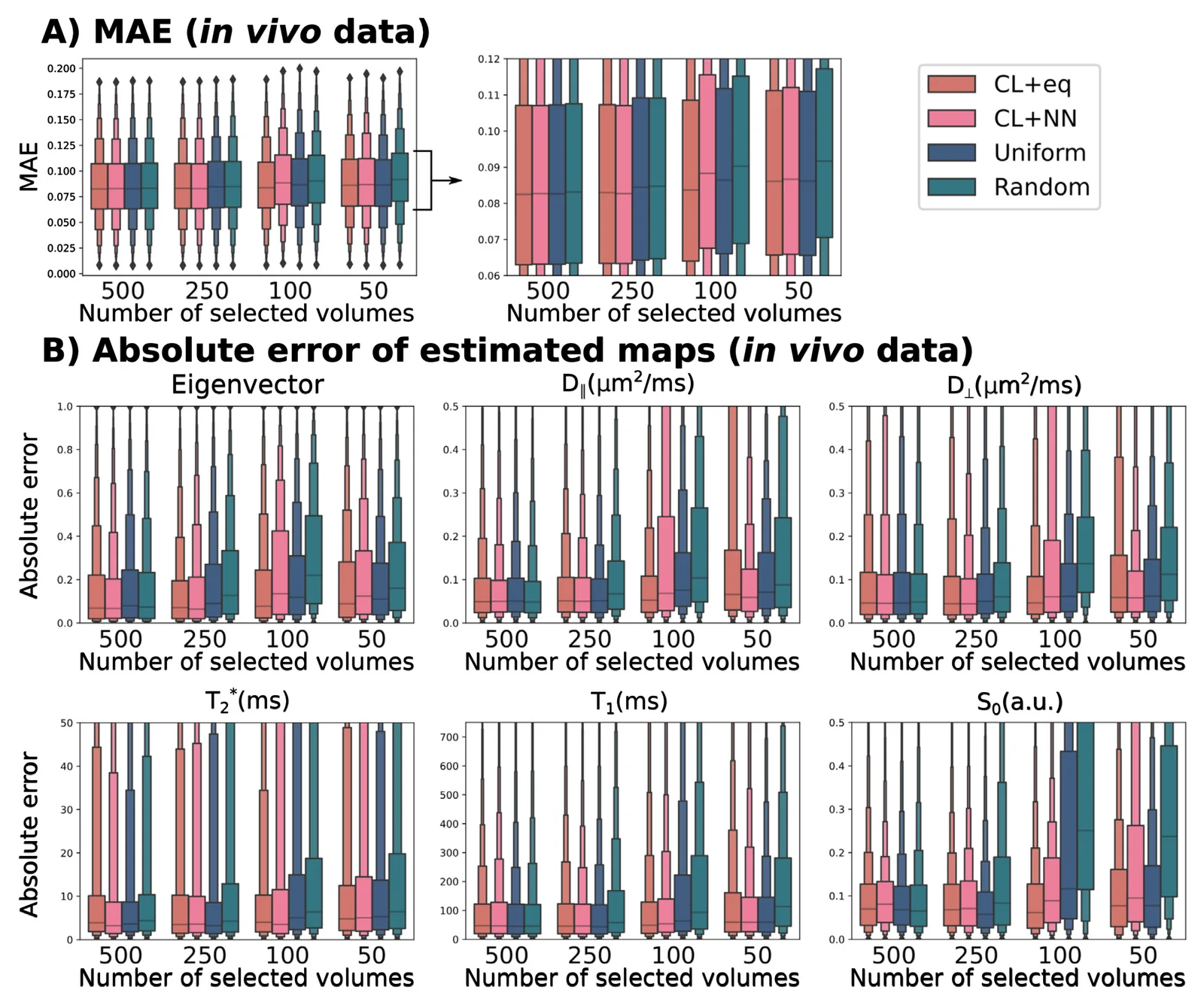
Comparison of the error of the predicted signal (A) and the estimated maps (B) from the test subject considering subject 3 as validation for four selection strategies according to the number of selected measurements with respect to the estimation obtained from the whole dataset. In (A), the left subfigure represents the whole range of mean absolute error (MAE) values, and the right subfigure is zoomed between 0.06 and 0.12. All the error values are shown for the whole brain. For the first eigenvector, the error was represented as one minus the dot product between the estimated and the ground truth maps to facilitate the interpretation. The highest errors have been omitted for visual purpose. A.u. = arbitrary units.

The MAE across the predicted signals with respect to the measured signals are shown in Fig. 6A. The MAE across methods was overall similar, with larger differences between ML and manual selection strategies for 100 and 50 selected volumes, and the Random selection having the largest MAE. CL+NN and the Uniform selection approach resulted in lower MAE for predictions of the non-selected measurements than the selected measurements (shown for *N* = 500 in Supplementary Figure 12 A), and the MAEs were similar regardless of the tissue type (Supplementary Figure 12B). The errors of the predicted signals across different validation subjects for *N* = 500 (Supplementary Table 7 and Supplementary Figure 12C) were similar for all the leave-one-out runs. CV and IQR values are presented in Supplementary Table 8, with the Random approach showing the lowest CV but the highest IQR values for *N* ≤ 250, and CL+NN and CL+eq the lowest IQR values for a high (*N* ≥ 250) and low (*N* < 250) number of sampled measurements, respectively.

#### 3.2.3 Parameter estimation

Fig. 6 shows the absolute error distributions of estimated maps with respect to estimation on all 1344 measurements. CL+eq showed consistently lower or similar median errors for all parameters and *N* with the exception of *S*_0_ and *N* = 250, where the median error and variability from the Uniform procedure were lower. Random selection showed the highest absolute errors, and for *N* < 250 the manual approaches show larger errors compared to the best performing CL+eq. Supplementary Figure 13 shows the spatial distribution of the errors in maps for each number of selected measurements.

## 4 Discussion

This study shows a proof-of-principle of using ML methods for the integrated selection of an optimal subset of MRI volumes, prediction of the MR signal, and estimation of quantitative parameters. Specifically, physics-informed networks incorporate a signal equation (either directly as in CL+eq or indirectly through predicting estimated parameters as in CL+par) to drive the sub-selection. The main finding of the work is that physics-informed NNs were able to select a subset of measurements that provide consistently accurate estimation of quantitative maps with CL+eq yielding the most similar estimates to those from all noisy measurements, especially in comparison with pure data-driven approaches and Random selection.

### 4.1 Sub-selection of measurements

#### 4.1.1 Variability in the selection

For all the ML selection procedures, physics-informed or data-driven, we assessed the stability of the distributions of the selected measurements across different initialisations and leave-one-out subjects for *in vivo* and synthetic data.

For *N* = 500 selected measurements, all ML selection methods showed stable results. Higher variability of the selected measurements was seen for different leave-one-out validations (Supplementary Figure 1) in comparison to different initialisations for a single subject (Fig. 2) for the physics-informed approaches (CL+eq and CL+par), but the main features of the distributions remained intact. A possible explanation for the variability is that measurements can correlate, e.g., for volumes with similar TIs, and hence there can be multiple subsets of selected measurements that contain the necessary information to similarly estimate maps and predict signals.

#### 4.1.2 Differences between ML-based selection methods

The main difference of the distributions between the physics-informed (CL+eq) and the pure data-driven (CL+NN) approaches, for both real and synthetic data, was the selection of the lowest SNR volumes with 500 selected submeasurements. The selection resulting from CL+eq presented a higher proportion of the lowest SNR volumes, i.e., the highest b-values (2000 and 3000 s/mm^2^) and lowest TI values (TI lower than 1000 ms). The increased selection of volumes with a higher SNR in the CL+NN approach is in line with the data-driven SARDU-Net selection (Grussu et al., 2021b), which consistently avoided the selection of the lowest SNR volumes. This may be explained by the relative importance of these volumes to predict the signal rather than estimate the quantitative maps. To predict the MRI signal, low SNR measurements may be less useful than others, but they may contain important information to characterise specific properties of biological tissues. Furthermore, the two physics-informed selection procedures CL+eq and CL+par showed similar selection results, although the proportion of lower SNR volumes was lower for the latter.

#### 4.1.3 Differences between the selection in synthetic and in vivo data

Regarding the comparison between the selection in synthetic and *in vivo* data with 500 measurements, the physics-informed selection showed similar distributions of the selected parameters, while the pure data-driven approach showed notable differences; i.e., fewer selected measurements with the highest SNR (b-values of 0 and 500 s/mm^2^) to increase the selection of low SNR volumes (b-values of 2000 and 3000 s/mm^2^ and TI values lower than 1000 ms). Differences between the *in vivo* and synthetic data include the noise distribution, presence of artifacts, and signal generation.

The noise distribution in the synthetic data was Gaussian whereas the noise distribution in *in vivo* magnitude data is typically Rician or non-central Chi distributed and may be (locally) altered by preprocessing. The reason for this choice was that the *in vivo* data was available and denoised in the complex domain and had relatively high SNR, reducing effects of the Rician noise floor (Gudbjartsson and Patz, 1995). Furthermore, with preprocessing strategies becoming more readily adopted to deal with the Rician noise (Tax et al., 2022), the current assessment is still representative. Finally, the primary aim was to address whether the proposed framework could make correct estimations of the quantitative parameters in the general presence of noise. Nevertheless, the influence of Rician noise, low SNR, and other loss functions than the L2 loss should be further explored.

The *in vivo* data contained some artifacts which may have affected the sampling. The synthetic data was generated based on a signal representation with one diffusion tensor, also for high b-values. However, kurtosis effects start to play a role at higher b-values due to e.g. microscopic kurtosis or multiple compartments. CL+NN may better capture this complexity as it is not restricted by assumptions on the signal equation. The framework can be extended with more complex signal representations accounting for these effects in future work.

#### 4.1.4 Selection on different tissue types

In this study, a whole-brain mask was used to train the selector layer, but the optimal selection of measurements most likely depends on tissue type. Training the selection procedure on WM and GM voxels separately in simulations did not result in significant improvement (results not shown); potentially because the same signal representation was used for both tissue types yet their parameter distribution was different. For *in vivo* data different biophysical models have been proposed for different tissue types, e.g. an isotropic tensor for CSF (Jelescu et al., 2016), intra-axonal sticks plus extra-axonal axially symmetric tensors for WM (Novikov et al., 2018, 2019) and additional contributions of a soma compartment and exchange for GM (Palombo et al., 2020; Jelescu et al., 2022). Physics-informed selection networks should therefore ideally be adapted to and trained on different tissue types, depending on the focus of a particular study. This could improve the estimation of quantitative maps and better characterise the microstructural properties of various tissues.

#### 4.1.5 Concrete selector

This work employs a concrete layer as selection layer, which can be regularised to select unique measurements, as carried out in this study. We hypothesised that as each parameter setting was included only once in the dataset (as opposed to repeated measurements), selecting measurements multiple times does theoretically not add new information, and it would be more informative to add new measurements. Yet, this may result in a higher loss compared to the selection without regularisation, but this was reduced by adopting very low values for the regularisation strength. However, the framework is fully flexible to tune the penalisation of repeated selection, and repeated selection could be an indication of further data redundancy (i.e. fewer measurements than the defined *N* are needed).

The CL has shown favourable results in predicting non-selected measurements in MRI (Pizzolato et al., 2020) and other tasks (Abid et al., 2019) compared to other selection techniques such as imposing (group) sparsity on the first layer. Nevertheless, the integrated selection and estimation approach presented here offers flexibility to implement other types of selection layers, e.g. the progressive subsampling suggested by Blumberg et al. (2022), which will be explored in future work. Furthermore, the proposed method was unable to provide recommendations on the acquisition of MRI measurements not included in the acquisition of the training dataset. Future work should explore the optimisation of acquisition parameters that are not necessarily a subset of the training data.

### 4.2 Prediction of signals and parameter estimation

The MAE of signal predictions was similar across methods for 500 and 250 measurements but higher for Random selections of 100 and 50 measurements.

Physics-informed approaches resulted more consistently in lower errors for quantitative maps in synthetic data and lower differences with estimates on the full dataset across parameters in synthetic experiments (quantified by the effect size) and *in vivo* data. Specifically, looking at the median error and its variability (IQR), an ML-based selection, mainly CL+eq, was usually among the two best methods, frequently presenting very small effect size. Yet, Uniform sampling performed relatively well with low estimation error and variability, especially for *N* 250 and 500. We hypothesise that this is because the initial set of all 1344 measurements was designed by experts and hence Uniform sub-sampling reflected this. Compared to CRLB, physics-informed selection (CL+eq) showed lower errors particularly for relaxation parameters, likely due to the *a priori* choice of tissue parameters in the CRLB approach and consequent narrow range of selected TIs. Both approaches incorporate a signal equation, but the CRLB approach relies on previous knowledge on tissue parameters (which is commonly set to a single value, in this study the mean of the ground truth distribution in the synthetic data), while the physics-informed approach implicitly considers the empirical distribution of the parameters. Moreover, the CL+par procedure minimises the error with respect to previously estimated parameters, and CL+eq explicitly considers no parameter estimates, but minimises the error with respect to the predicted signal. The need for detailed *a priori* knowledge, considering different tissue types, makes an optimal extraction of the acquisition parameters difficult in comparison with an empirical approach such as the physics-informed framework proposed in this work. Furthermore, noise may have influenced the results, considering that CRLB *a priori* parameter values were estimated from ground truth maps. Therefore, physics-informed learning seems an appropriate approach for diffusion-relaxation MRI experiment design providing reliable results for distributions of multi-contrast parameters.

#### 4.2.1 Network for the evaluation of sub-selections

To evaluate the sub-protocols from different selection methods, a separate network for each subset was trained with a similar structure to the decoder of the CL+eq approach (Fig. 1A), providing predictions of the signals as well as estimated maps by integrating a signal equation (Hutter et al., 2018; Tax et al., 2021a). This network was kept consistent between selection procedures to assess the effect of the selected measurements only. However, the concrete selection layer of each approach was trained in conjunction with their specific decoder, and hence training a network similar to this decoder for the evaluation of the sub-selection may result in lower errors. For example, to predict the MR signal without necessarily estimating parameter maps, non-physics informed methods (e.g. a fully-connected NN directly predicting the signal) could provide more accurate values as they may be able to better represent higher complexity at the cost of potential overfitting. On the other hand, physics-informed or model-based approaches can be used to simultaneously estimate quantitative maps, which is often the eventual goal when analysing data, at the cost of losing accuracy for the prediction of the signal.

#### 4.2.2 Considerations on the estimation of quantitative parameters

The quantitative estimates showed biases (i.e. non-zero errors compared to the ground truth) potentially related to local minima, particularly for a low number of selected volumes. Here we have used both ML-based estimators as well as NLS. The use of ML for the estimation of quantitative maps has received increased attention recently, particularly in the area of dMRI with its many degrees of freedom (Nedjati-Gilani et al., 2017; Reisert et al., 2017; de Almeida Martins et al., 2021a; Palombo et al., 2020).

Most ML-based estimation work focused on estimation from full protocols and were either based on supervised and self-supervised strategies, which inspired the CL+par and CL+eq approaches, respectively (Grussu et al., 2021a). Supervised approaches rely on training data with known outputs (i.e. estimated maps), and for dMRI estimation Random forest (Nedjati-Gilani et al., 2017) and NNs have been employed, amongst others. Several studies have raised awareness regarding potential caveats in supervised NN estimation. Specifically, estimation can be significantly impacted by the training data distribution. This work used a distribution reflecting healthy brain, but other choices could be made, e.g. uniform distributions or a mix, each with their advantages and disadvantages such as biases in atypical parameter combinations not present in the training set (Gyori et al., 2022). In most cases the approach is trained on synthetic data with added noise reflecting the *in vivo* data, but it could also be trained on estimates from *in vivo* data as silver standard, in which case the approach may become dependent on the estimator. In contrast to most previous works, this work does not take the directional average which may provide richer information and reduce bias. Compared to random forest regression, Gyori et al. (2022) showed lower variability yet lower accuracy for NNs, but NNs could be slower and potentially present higher risk of local minima than regression methods (Coelho et al., 2022). Gyori et al. (2022) found better accuracy but lower precision of NLS estimates compared to NN-based estimates, especially when reducing the SNR.

Grussu et al. (2021a) compared supervised and self-supervised NN strategies for the estimation of quantitative maps and found that the former leads to lower tissue parameter MSE in the case of Rician noise, whereas the latter results in lower predicted signal MSE. Kaandorp et al. (2021) used unsupervised physics-informed deep learning to estimate rotationally invariant intravoxel incoherent motion (IVIM) parameters in simulated and *in vivo* data from pancreatic cancer patients. They showed in simulated data lower estimation error of the parameters for NN considering low SNR (SNR < 50), but not for high SNR, and similar values compared to LS and Bayesian approaches in *in vivo* data. Lim et al. (2022) included the estimation of orientational features and showed better estimation of the parameters from the *T*_1_-ball-stick model using a NN compared to NLS in synthetic and *in vivo* data considering 416 volumes of the MUDI dataset.

In the present study generally a self-supervised strategy was adopted for estimation of both rotationally invariant and orientational features, with the advantage that it does not require training on synthetic data and could in theory handle spatial variations in acquisition parameters, e.g. in the case of slice-interleaving (Hutter et al., 2018) or gradient non-uniformities (Bammer et al., 2003). The use of a supervised strategy (e.g. based on simulations or previously obtained estimates on data such as in the decoder of CL+par) could improve the estimations of the parameters, particularly for Rician noise (Grussu et al., 2021a). However, the normalisation to avoid a higher weight of the parameters with the highest values (*T*_1_ and$T_{2}^{*}$) may influence the results. A possible solution is to use a loss function incorporating the CRLB (Zhang et al., 2022). Specifically, a normalisation of the squared error for each quantitative parameter estimated with NNs was proposed to get a minimum variance unbiased estimator and avoid a major effect of parameters that are difficult to predict. The self-supervised approach intrinsically adjusts the weights for each parameter and, possibly, the proper adjustment of the components with higher importance on the values of the signal may also contribute to a better estimation of parameters that explain a lower percentage of the variance of the signal. A supervised selection approach (CL+par) was only implemented for synthetic data. It could also be implemented for *in vivo* data with the estimates from the full dataset as output.

Regarding the comparison with NLS estimation, we observed that the ML estimated quantitative maps showed higher error and variability in general for any number of selected measurements, especially in WM and non-diffusion related parameters (*T*_1_, $T_{2}^{*}$ and *S*_0_). Compared to other work (Lim et al., 2022), the NNs employed for the estimation presented a similar architecture but were less complex, i.e., the number of hidden layers was lower to match the decoder in the CL+eq selection network, which could be a key factor of the different results in unseen data. Furthermore, the use of data augmentation techniques may contribute to improve the estimation results.

In this study, as expected, we obtained estimates with lower error values when training on synthetic compared to *in vivo* data. Apart from the difference between *in vivo* data and model fitting, a reason of the difference may be related to the complexity of the NN. Gyori et al. (2022) showed that more complex network architectures are necessary to map parameters from noise-free data, and in this work the complexity of the architecture was equal for data with and without noise, i.e., synthetic and *in vivo* volumes, respectively. Additionally, ML methods, particularly NNs, estimate smoothed maps, as shown in Gyori et al. (2022), which would justify the lower variability but higher bias of these methods compared to NLS. In our case, potentially due to the relatively simple architecture of the NNs, higher variability and error was observed using ML to estimate the quantitative maps. Thus, it is also important to consider whether training is carried out with preprocessed or raw volumes, as the architecture employed for a specific type of data may differ according to the nature of the input data.

Previous studies employing ML techniques have assessed the effect of reducing the number of MRI volumes and different sampling strategies (Merlet et al., 2013; Golkov et al., 2016; de Almeida Martins et al., 2021a; Grussu et al., 2021b; Chen et al., 2020; Tian et al., 2020). When reducing the number of acquired volumes, it has been observed that the estimation of the diffusion direction was more sensitive to noise (Merlet et al., 2013), but the estimation of other parameters with NNs was less affected by suboptimal protocols in comparison with least-squares approaches (de Almeida Martins et al., 2021a) or acquisition subprotocols not based on ML selection (Grussu et al., 2021b). Golkov et al. (2016) achieved a similar or even better estimation with 12 out of 158 MRI volumes using Deep Learning compared to the standard fitting estimation using the full dataset for kurtosis and NODDI parameters. Here, we show a proof-of-principle of the joint optimization of a reduced acquisition, the prediction of non-selected measurements, and/or the estimation of quantitative maps, instead of applying these steps separately, and obtaining comparable results to the whole dataset when highly reducing the number of employed volumes. Therefore, the results from our study suggest that an intelligent sampling optimised for the employed models may be beneficial not only to reduce the MRI acquisition time to one adequate for clinical routine, but also to retrieve the full potential of the data for the characterisation of brain microstructure.

### 4.3 Other limitations and future work

#### 4.3.1 Generalisability

The current study performed extensive evaluation on a publicly-available diffusion-relaxation dataset. Nevertheless, future work should explore generalisability to datasets from other organs or acquired with other encoding techniques (e.g. beyond linear tensor encoding) to optimise the selection of efficient protocols and estimation of quantitative maps (Szczepankiewicz et al., 2021). Previous work has evaluated the pure data-driven approach (CL+NN) on a different 5D dataset that varied b-value, TE, gradient direction, and diffusion encoding waveform (*b*_*Δ*_) (Tax et al., 2021b). The physics-informed approaches require prior knowledge on the model and the set of acquisition parameters and can be adapted accordingly. Although this work focused on the optimisation of diffusion-relaxation protocols for which the dimensionality of the acquisition-parameter space becomes exceedingly large compared to diffusion or relaxation MRI alone, the method can easily be adapted to further shorten protocols for diffusion or relaxation MRI currently used in clinical research studies.

The simulations in this study used the same simplified signal equation to generate ground truth signals as to estimate parameters, to be able to assess accuracy. Consequently, there were effects that were not considered such as partial volume, IVIM or kurtosis with a potential effect on the estimated parameters and predicted MRI signal. Future work could consider more realistic simulations including multiple compartments or Monte Carlo simulations in realistic meshes.

The total number of subjects for this proof-of-concept, five for training and validation and another one for testing, was relatively low due to the acquisition time, which could limit the generalisability of the results as the distribution of a healthy population cannot fully be captured. Nevertheless, as each voxel is considered as a training example, the network can leverage the wide range of microstructural configurations throughout the brain. Additionally, only data from healthy subjects were assessed, and future work could include data from various neurological or psychiatric disorders.

#### 4.3.2 Hyperparameter optimisation

The use of concrete autoencoders showed promising results, but the risk of local minima should be considered. Temperature and regularisation hyperparameters in this work were chosen based on previous work, and the number of layers in the selection networks was based on an assessment of up to three layers of the original CL+NN approach (Tax et al., 2021b), with the validation error being lowest for the used setting. The estimation network was set to match the decoder of CL+eq, but hyperparameters can be further optimised in future work. Finally, the current approach does not incorporate rotational invariance.

## 5 Conclusion

The present approach combining ML feature selection with physics-informed signal modelling can exploit MRI data redundancy to estimate non-acquired data and quantitative parameters from reduced datasets more suitable for clinical research studies. Non-acquired data could be predicted and quantitative parameters estimated with a 5-fold reduced protocol, yielding the most consistently very small effect size and hence similar error-distributions compared to the full protocol across the evaluated methods. Manual Uniform sampling can perform well particularly for a high number of sampled measurements (*N* ≥ 250), presumably because it reflects the expert design of the full protocol. This framework can be extended for the selection of optimal sets of acquisition parameters for a wide range of MRI sequences and tissue types.

## Supplementary Material

Supplementary Material

## Figures and Tables

**Table 1:** Main changes of the subsampling strategy of *N* for synthetic data

Selection	500 to 250	250 to 100	100 to 50
CL+eq	↓ b = 3000 s/mm^2^ ↓ medium-high TI (b = 2000 s/mm^2^)	↓ b = 0 s/mm^2^ ↓ low-medium TI (b = 500; 1000; 2000 s/mm^2^)	↓ b = 500; 1000 2000 s/mm^2^
CL+NN	↓ b = 3000 s/mm^2^ ↓ medium TI (b = 2000 s/mm^2^) ↓ b = 500 s/mm^2^ (TD = 50 ms)	↓ b ≤ 500 s/mm^2^ ↓ medium-high TI (b ≥ 1000 s/mm^2^)	↓ b = 1000; 2000 s/mm^2^
CL+par	↓ b ≤ 500 s/mm^2^ ↓ TD = 50 ms (b ≥ 1000 s/mm^2^)	↓ medium TI (b ≥ 1000 s/mm^2^)	↓ b ≥ 1000 s/mm^2^

*↓ = reduced sampling*.

**Table 2:** Median values of the mean absolute error (MAE) values of the predicted MRI signal and the estimated quantitative parameters on a test subject depending on the number of selected measurements for synthetic data. a.u. = arbitrary units. The error obtained for the estimation with the full dataset has been included (*E*_1344_). The error for the first eigenvector was shown as 1 minus the dot product between the ground truth and the estimated eigenvector to facilitate the interpretation of the results. The two best results are marked in yellow, and the two worst in magenta.

Parameter		Selection	N = 500	N = 250	N = 100	N = 50

Signal MAE (a.u.)		CL+par	0.051	0.052	0.053	0.055
		CL+eq	0.051	0.052	0.052	0.055
		CL+NN	0.051	0.051	0.053	0.055
		Uniform	0.052	0.052	0.053	0.055
		Random	0.051	0.053	0.057	0.058
		CRLB	0.052	0.051	0.053	0.054
*D*_∥_ Error (*μm*^2^/*m5*) *E*_1344_ = 0.015		CL+par	**0.016**	0.018	0.024	0.034
		CL+eq	0.017	0.018	**0.023**	0.036
		CL+NN	0.020	0.017	0.026	0.033
		Uniform	0.018	0.019	0.025	0.037
		Random	0.018	0.024	0.025	0.047
		CRLB	0.016	**0.016**	0.034	**0.030**
*D_⊥_* Error *(μm*^2^*/ms)* *E*_1344_ = 0.022		CL+par	**0.018**	0.022	**0.028**	0.051
		CL+eq	0.020	0.024	0.029	0.044
		CL+NN	0.023	0.022	0.032	0.046
		Uniform	0.022	0.026	0.035	0.047
		Random	0.020	0.030	0.086	0.058
		CRLB	0.022	**0.020**	0.034	**0.041**
$T_{2}^{*}$ Error (ms) *E*_1344_ = 1.248		CL+par	1.337	1.972	2.591	3.688
		CL+eq	**1.037**	1.232	1.981	2.604
		CL+NN	1.605	1.640	2.098	2.689
		Uniform	1.444	**1.149**	**1.705**	**2.411**
		Random	1.221	1.811	2.956	4.705
		CRLB	1.379	1.445	2.083	2.595
*T_1_* Error (ms) *E*_1344_ = 10.903		CL+par	16.134	21.359	33.590	29.915
		CL+eq	**12.535**	**13.839**	**20.145**	**29.236**
		CL+NN	16.995	15.226	24.850	29.699
		Uniform	14.269	16.628	23.261	31.678
		Random	16.642	24.116	36.354	54.682
		CRLB	14.330	16.262	25.064	35.239
*S*_0_ Error (a.u.) *E*_1344_ = 0.016		CL+par	0.029	0.035	0.044	0.040
		CL+eq	0.019	**0.019**	0.036	**0.037**
		CL+NN	0.043	0.030	0.043	0.045
		Uniform	0.018	0.021	**0.031**	0.041
		Random	0.025	0.038	0.055	0.095
		CRLB	**0.017**	0.020	0.034	0.041
Eigenvector (1 - dot product) *E*_1344_ = 0.007		CL+par	**0.006**	0.011	0.023	0.028
		CL+eq	0.008	0.012	0.017	0.037
		CL+NN	0.009	**0.009**	0.025	0.036
		Uniform	0.011	0.016	0.019	0.034
		Random	0.009	0.027	0.025	0.047
		CRLB	0.009	0.010	**0.011**	**0.022**

a.u. = arbitrary units.

**Table 3:** Main changes of the subsampling strategy for each reduction step of the number selected measurements for *in* *vivo* data.

Selection	500 to 250	250 to 100	100 to 50
CL+eq	↓ low TI (b ≥ 1000 s/mm^2^) Uniform reduction	↓ low-medium TI (b ≥ 500 s/mm^2^)	↓ medium-high TI Uniform reduction
CL+NN	↓ low-medium TI (b ≥ 1000 s/mm^2^) Uniform reduction	↓ medium-high TI (b ≥ 1000 s/mm^2^)	↓ low-medium TI (b ≥ 500 s/mm^2^)

↓= reduced sampling.

## Data Availability

The data used in this study were provided by the organisers of the MUDI challenge.

## Code availability statement

The code employed for the selection and estimation networks is publicly available at: https://github.com/aplanchu/qMRI-CAO
