# Supplementary material for: Optimisation of quantitative brain diffusion-relaxation MRI acquisition protocols with physics-informed machine learning
Source: Med Image Anal. Author manuscript; Available in PMC 2026 Nov 7. (PMC7619710; doi:10.1016/j.media.2024.103134)
Supplement: Supplementary file 1 [file EMS221413-supplement-Supplementary_Material.pdf]

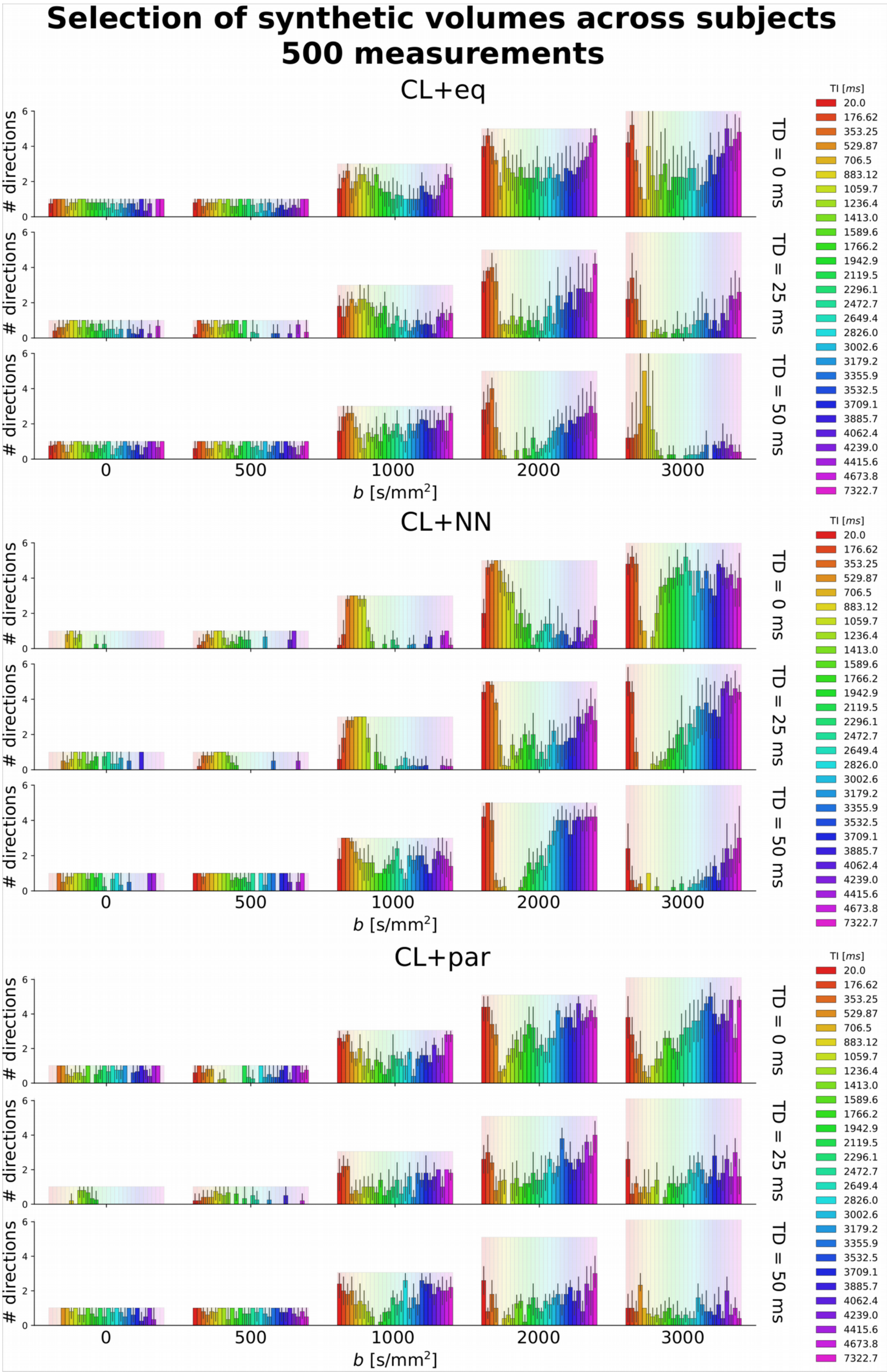

Supplementary Figure 1. Comparison of the selected 500 measurements between the machine learning methods. The histograms represent the mean number of chosen volumes (gradient orientations) for each b-value (0, 500, 1000, 2000 and 3000 s/mm²),

TD (0, 25 and 50 ms), and TI (28 values from 20 to 7322.7 ms) across five different validation subjects. The errorbars represent the standard deviation.

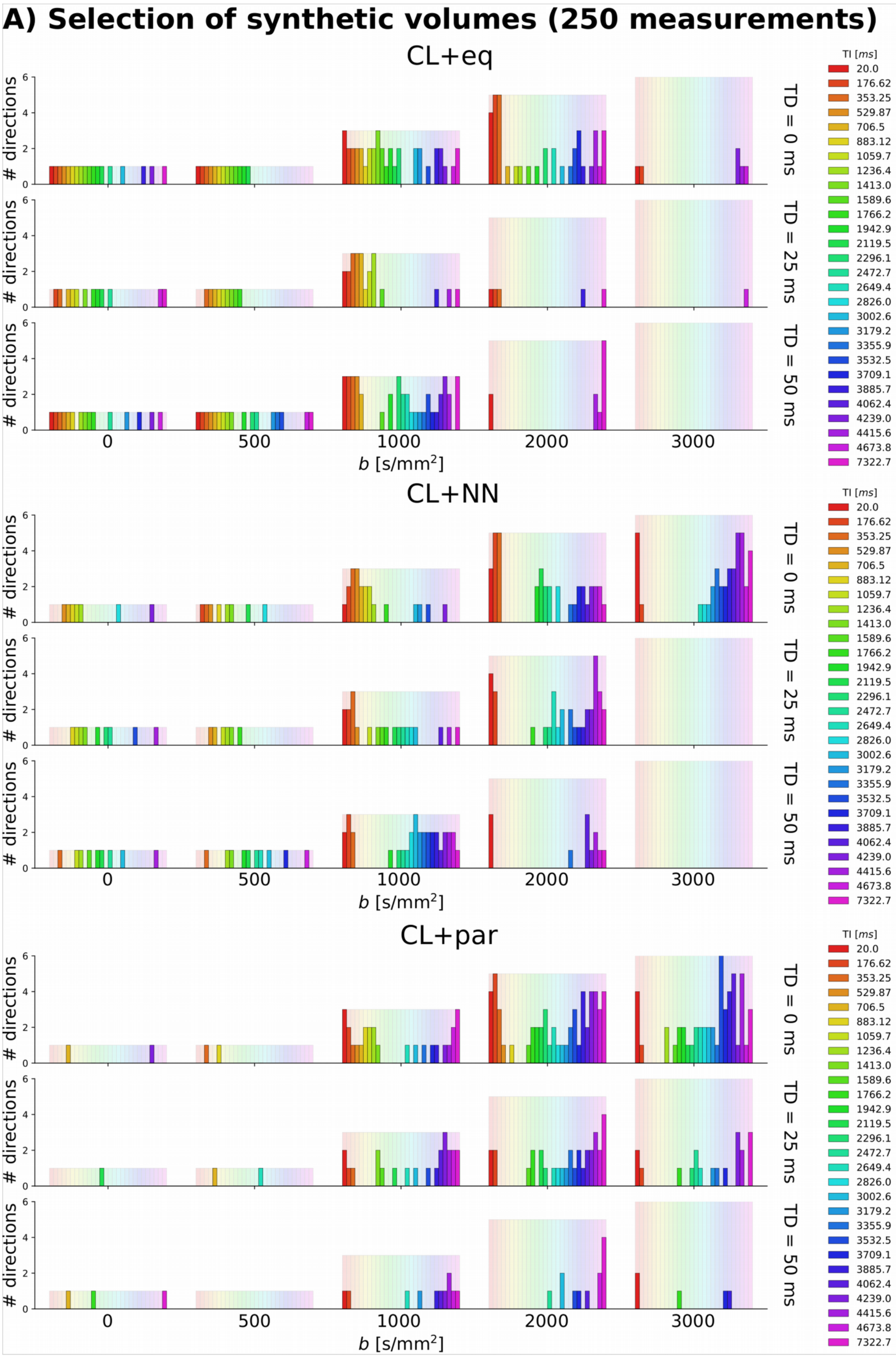
A) Selection of synthetic volumes (250 measurements)
CL+eq
CL+NN
CL+par
# directions
TD = 0 ms
TD = 25 ms
TD = 50 ms
0
500
1000
2000
3000
b [s/mm²]
TI [ms]
20.0
176.62
353.25
529.87
706.5
883.12
1059.7
1236.4
1413.0
1589.6
1766.2
1942.9
2119.5
2296.1
2472.7
2649.4
2826.0
3002.6
3179.2
3355.9
3532.5
3709.1
3885.7
4062.4
4239.0
4415.6
4673.8
7322.7

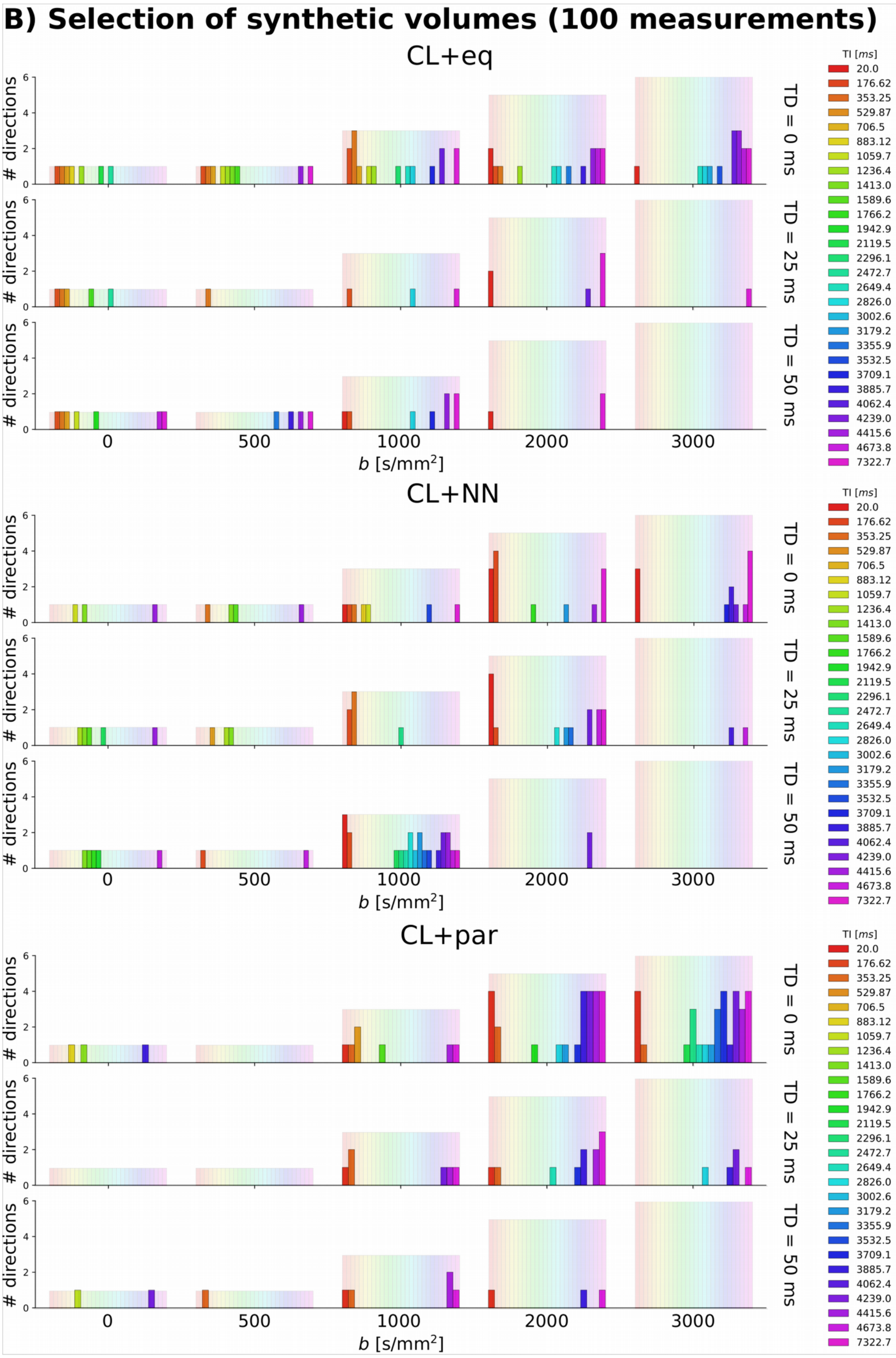
B) Selection of synthetic volumes (100 measurements)
CL+eq
CL+NN
CL+par
# directions
TD = 0 ms
TD = 25 ms
TD = 50 ms
0
500
1000
2000
3000
b [s/mm^2]
TI [ms]
20.0
176.62
353.25
529.87
706.5
883.12
1059.7
1236.4
1413.0
1589.6
1766.2
1942.9
2119.5
2296.1
2472.7
2649.4
2826.0
3002.6
3179.2
3355.9
3532.5
3709.1
3885.7
4062.4
4239.0
4415.6
4673.8
7322.7

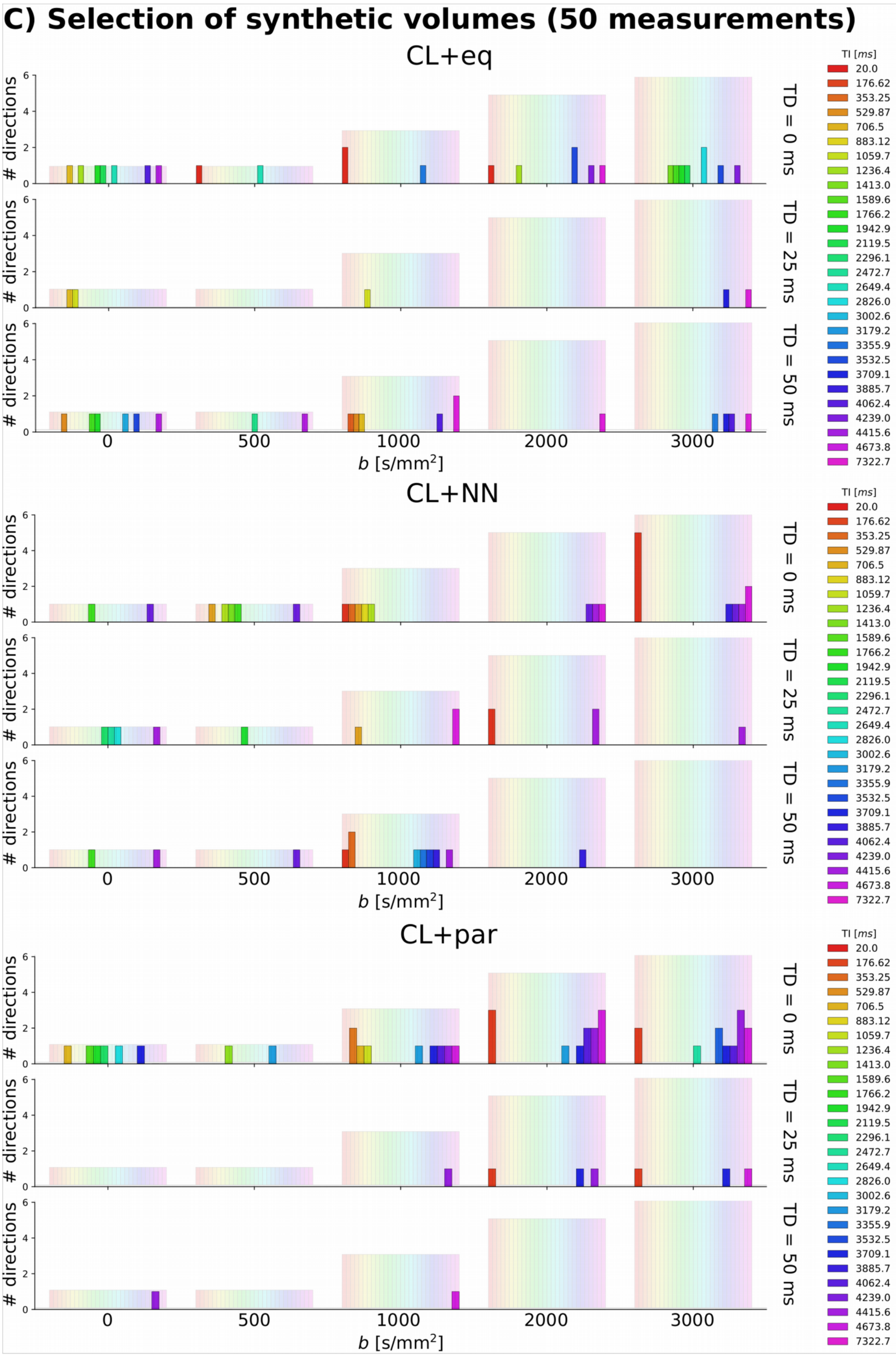

Supplementary Figure 2. Comparison of the selected 250 (A), 100 (B) and 50 (C) measurements between the machine learning selection methods. The histograms represent the mean number of chosen volumes (gradient orientations) for each b-value (0, 500, 1000, 2000 and 3000 s/mm²), TD (0, 25 and 50 ms), and TI (28 values from 20 to 7322.7 ms) for one validation subject.

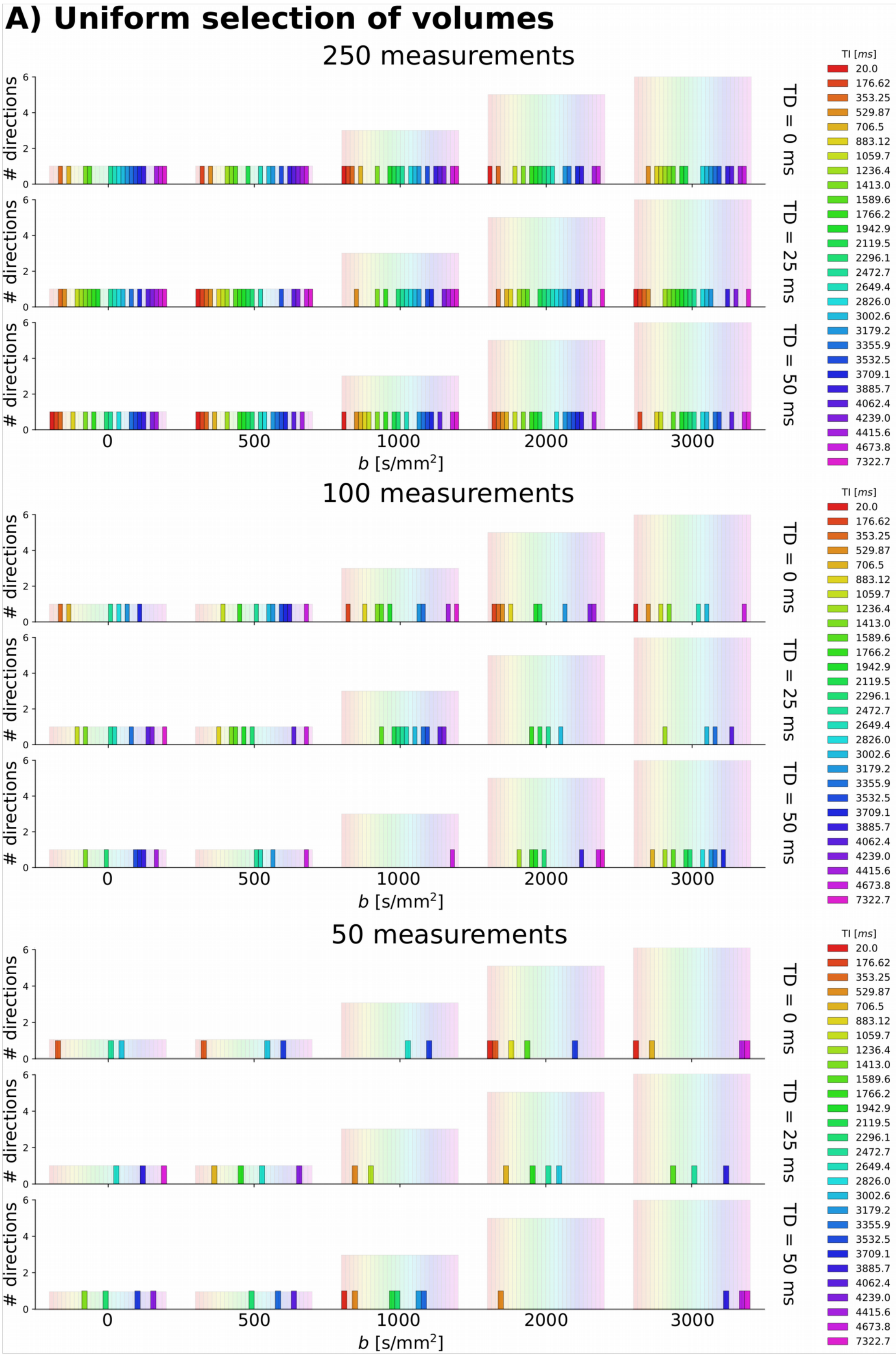
A) Uniform selection of volumes
250 measurements
100 measurements
50 measurements
# directions
TD = 0 ms
TD = 25 ms
TD = 50 ms
0
500
1000
2000
3000
b [s/mm²]
TI [ms]
20.0
176.62
353.25
529.87
706.5
883.12
1059.7
1236.4
1413.0
1589.6
1766.2
1942.9
2119.5
2296.1
2472.7
2649.4
2826.0
3002.6
3179.2
3355.9
3532.5
3709.1
3885.7
4062.4
4239.0
4415.6
4673.8
7322.7

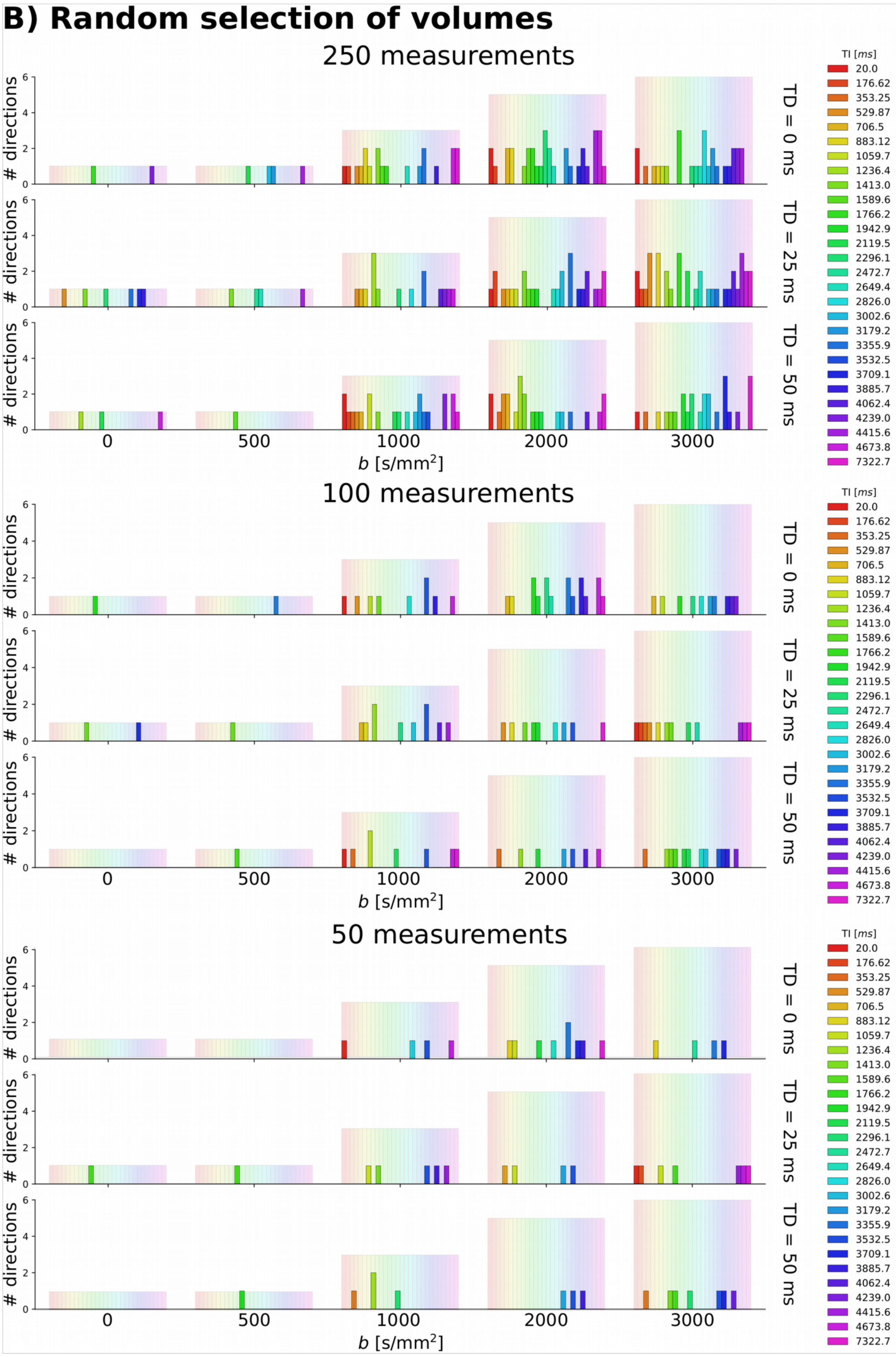

Supplementary Figure 3. Selected measurements of the Uniform (A) and Random (B) manual selection procedures for protocol size of 250, 100 and 50 volumes. The histograms represent the mean number of chosen volumes (gradient orientations) for each b-value (0, 500, 1000, 2000 and 3000 s/mm²), TD (0, 25 and 50 ms), and TI (28 values from 20 to 7322.7 ms). The same distribution was used for all the subjects.

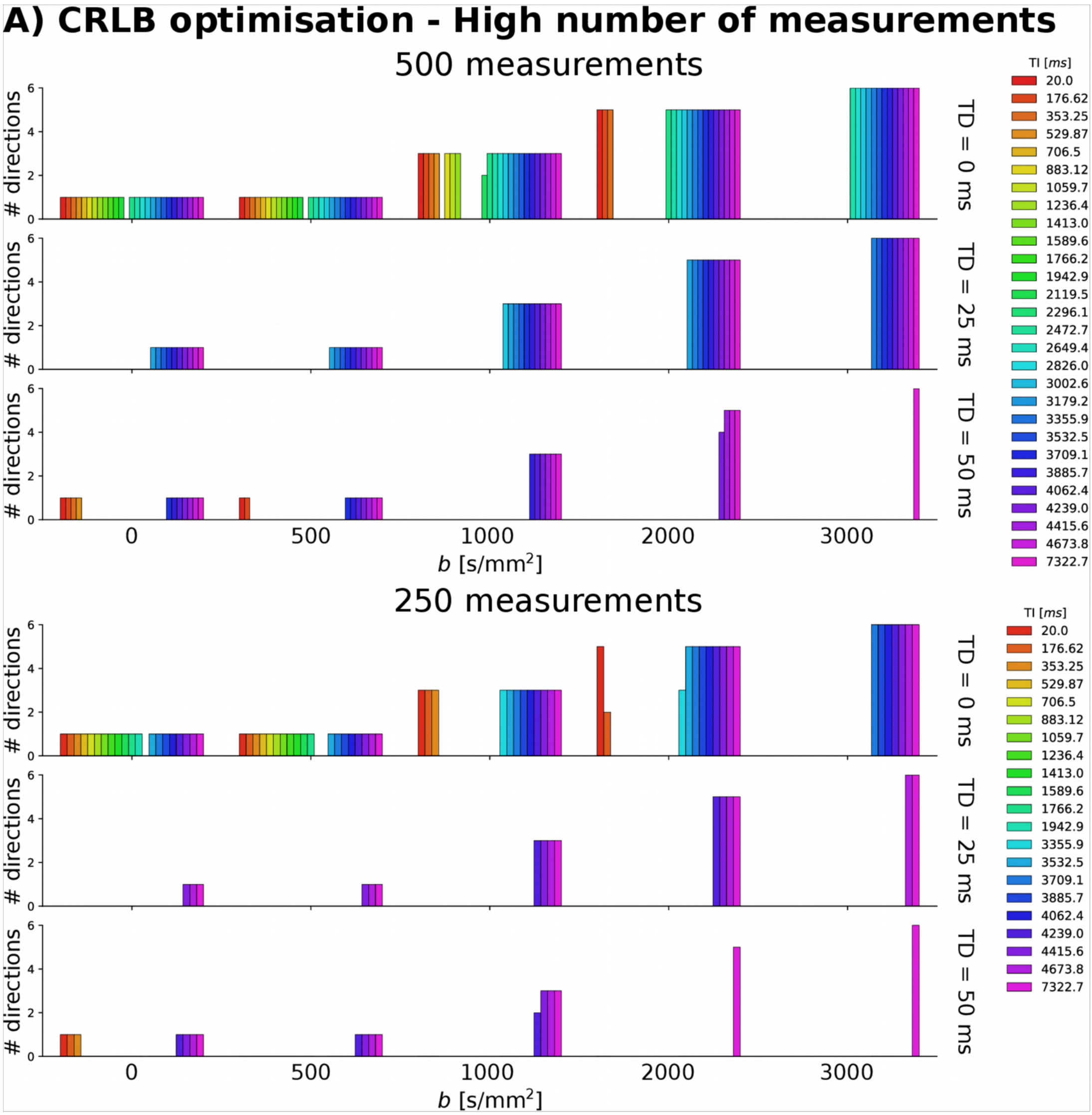
A) CRLB optimisation - High number of measurements
500 measurements
# directions
TD = 0 ms
TD = 25 ms
TD = 50 ms
0
500
1000
2000
3000
b [s/mm²]
TI [ms]
20.0
176.62
353.25
529.87
706.5
883.12
1059.7
1236.4
1413.0
1589.6
1766.2
1942.9
2119.5
2296.1
2472.7
2649.4
2826.0
3002.6
3179.2
3355.9
3532.5
3709.1
3885.7
4062.4
4239.0
4415.6
4673.8
7322.7
250 measurements
# directions
TD = 0 ms
TD = 25 ms
TD = 50 ms
0
500
1000
2000
3000
b [s/mm²]
TI [ms]
20.0
176.62
353.25
529.87
706.5
883.12
1059.7
1236.4
1413.0
1589.6
1766.2
1942.9
3355.9
3532.5
3709.1
3885.7
4062.4
4239.0
4415.6
4673.8
7322.7

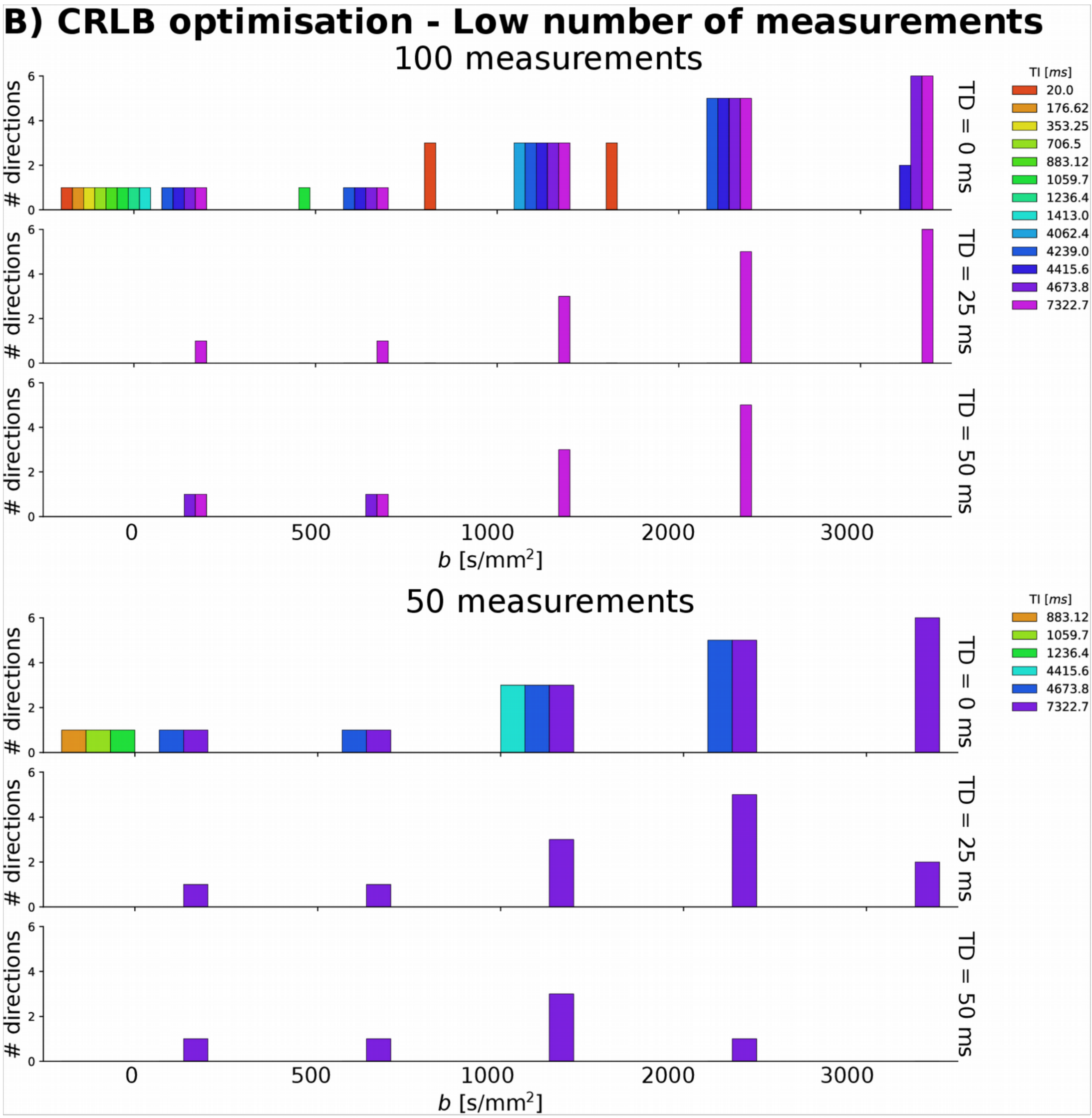

Supplementary Figure 4. Comparison of the schemes with selected measurements using the optimisation of the Fisher matrix information and the Cramér-Rao Lower Bound (CRLB) for: A) Highest number of selected measurements (500 and 250); B) Lowest number of selected measurements (100 and 50). The histograms represent the chosen volumes (gradient orientations) for each b-value (0, 500, 1000, 2000 and 3000 s/mm²), TD (0, 25 and 50 ms), and TI (28 values from 20 to 7322.7 ms).

Supplementary Table 1. Median values of the mean absolute error (MAE) values of the predicted MRI signal and the estimated quantitative parameters on a test subject depending on the validation subject for synthetic data for 500 selected measurements. The two best results are marked in yellow.

	Selection	Subj. 1	Subj. 2	Subj. 3	Subj. 4	Subj. 5
Signal MAE (a.u.)	CL+par	0.051	0.051	0.051	0.051	0.051
	CL+eq	0.051	0.051	0.051	0.051	0.051
	CL+NN	0.052	0.052	0.051	0.052	0.051
	Uniform	0.051	0.051	0.052	0.051	0.051
	Random	0.051	0.051	0.053	0.051	0.051
$D_{\parallel}$ AE ($\mu m^2/ms$)	CL+par	**0.014**	**0.016**	**0.016**	0.017	**0.015**
	CL+eq	0.015	0.017	0.017	**0.015**	0.015
	CL+NN	0.018	0.020	0.020	0.018	0.017
	Uniform	0.018	0.017	0.018	0.018	0.022
	Random	0.017	0.018	0.018	0.018	0.016
$D_{\perp}$ AE ($\mu m^2/ms$)	CL+par	**0.020**	**0.018**	**0.014**	0.020	**0.018**
	CL+eq	0.022	0.021	0.015	**0.019**	0.019
	CL+NN	0.022	0.022	0.017	0.021	0.020
	Uniform	0.024	0.022	0.016	0.023	0.026
	Random	0.020	0.019	0.015	0.019	0.019
T_2^* AE (ms)	CL+par	1.439	1.251	1.337	1.158	1.199
	CL+eq	**1.158**	1.148	**1.037**	1.341	1.106
	CL+NN	1.926	1.989	1.605	1.705	1.358
	Uniform	1.199	**1.005**	1.444	**1.009**	**1.048**
	Random	1.303	1.261	1.221	1.341	1.389
T_1 AE (ms)	CL+par	**14.818**	17.647	16.134	**14.108**	**13.878**
	CL+eq	15.073	14.208	**12.535**	15.239	14.194
	CL+NN	17.775	16.965	16.995	18.158	15.503
	Uniform	18.521	**14.145**	14.249	14.153	14.890
	Random	16.318	17.524	16.642	17.003	16.109
S_0 AE (a.u.)	CL+par	**0.019**	0.026	0.013	**0.020**	**0.020**
	CL+eq	0.021	0.020	0.008	0.022	0.023
	CL+NN	0.052	0.045	0.018	0.046	0.035
	Uniform	0.023	**0.018**	**0.008**	0.022	0.024
	Random	0.022	0.024	0.011	0.024	0.023
Eigenvector (1-dot product)	CL+par	**0.007**	**0.007**	**0.006**	0.008	0.008
	CL+eq	0.009	0.010	0.008	**0.008**	**0.007**
	CL+NN	0.009	0.012	0.009	0.010	0.009
	Uniform	0.010	0.009	0.011	0.009	0.011
	Random	0.011	0.008	0.009	0.008	0.011

a.u. = arbitrary units. The error for the first eigenvector was shown as 1 minus the dot product between the ground truth and the estimated eigenvector to facilitate the interpretation of the results.

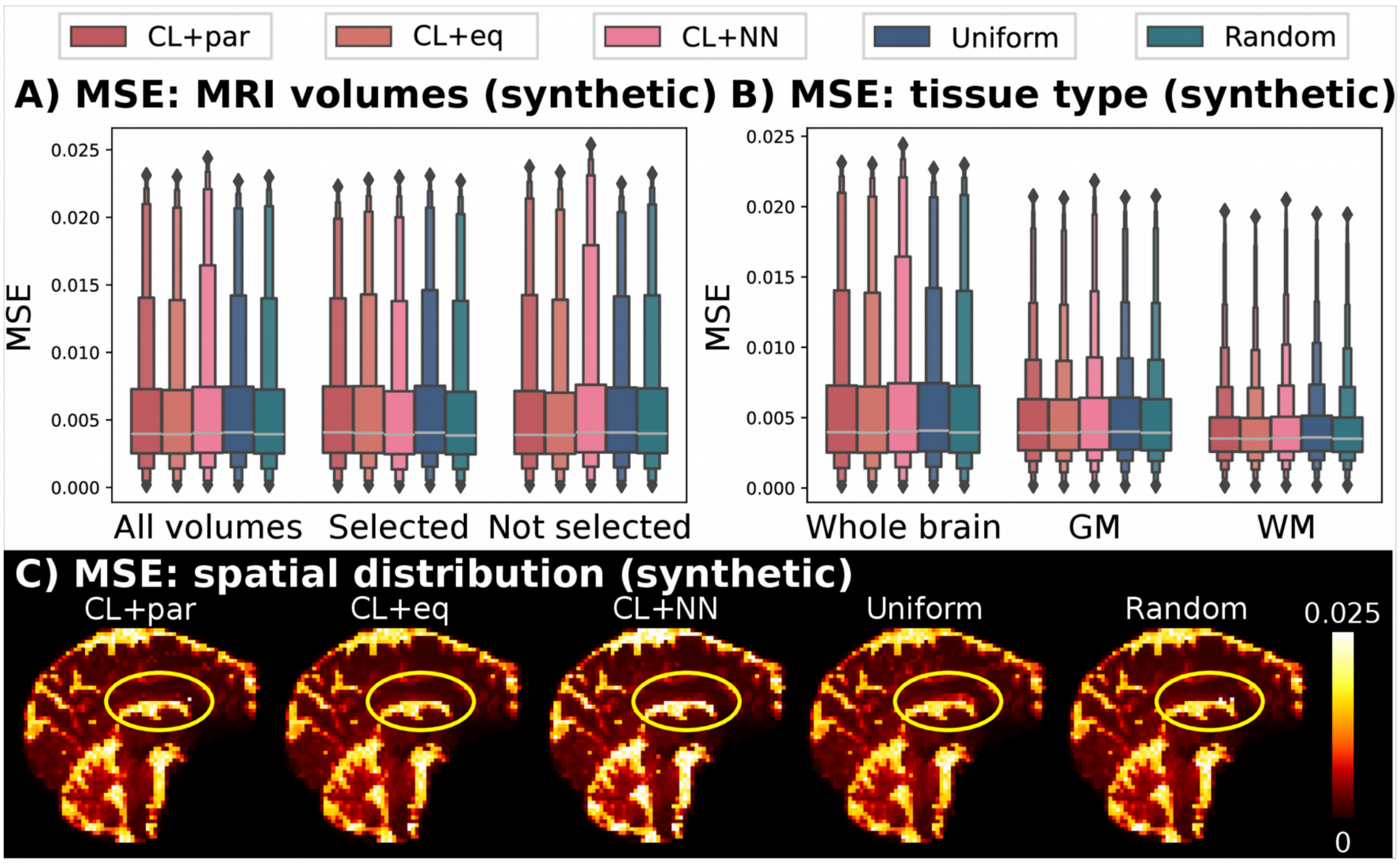

Supplementary Figure 5. Mean squared error (MSE) of the predicted synthetic measurements from the test subject in one of the cross-validation procedures for five selection strategies and 500 selected submeasurements subdivided by: A) MRI volumes (whole dataset, and volumes used and unused to train the estimation network); B) Tissue type (whole brain, grey matter-GM, and white matter-WM). C) Spatial distribution of the MSE for all the volumes. The yellow ellipses show an area around the cingulum, where prediction differences between methods can be appreciated, showing the predictions from the physics-informed selection lower error values.

Supplementary Table 2. Mean squared error (MSE), and coefficient of variation (CV) and interquartile range (IQR) of the MSE of the predicted signal depending on the volumes in relation to training and the main brain tissue types from synthetic data of the test subject considering synthetic subject 3 as validation.

Selection (N = 500)	All volumes	Selected volumes	Non-selected volumes	Grey matter (all volumes)	White matter (all volumes)
**MSE**					
CL+par	0.0071	0.0071	0.0072	0.0061	0.0053
CL+eq	0.0071	0.0072	0.0070	0.0060	0.0052
CL+NN	0.0074	0.0070	0.0077	0.0063	0.0054
Uniform	0.0072	0.0073	0.0071	0.0061	0.0054
Random	0.0071	0.0069	0.0072	0.0061	0.0053
**CV**					
CL+par	0.9998	**0.9688**	1.0226	0.9233	0.9513
CL+eq	0.9996	0.9928	1.0111	**0.9177**	**0.9452**
CL+NN	1.0184	1.0030	1.0324	0.9456	0.9774
Uniform	**0.9815**	0.9973	0.9779	0.9188	0.9613
Random	0.9980	1.0037	**0.9973**	0.9180	0.9505
**IQR**					
CL+par	0.0056 [0.0026, 0.0083]	0.0059 [0.0026, 0.0085]	0.0056 [0.0026, 0.0081]	0.0043 [0.0028, 0.0070]	0.0028 [0.0026, 0.0054]
CL+eq	0.0056 [0.0026, 0.0082]	0.0059 [0.0026, 0.0086]	0.0055 [0.0026, 0.0080]	0.0042 [0.0027, 0.0070]	0.0028 [0.0026, 0.0054]
CL+NN	0.0058 [0.0026, 0.0085]	0.0056 [0.0025, 0.0082]	0.0060 [0.0027, 0.0087]	0.0044 [0.0028, 0.0071]	0.0028 [0.0027, 0.0055]
Uniform	0.0058 [0.0027, 0.0085]	0.0060 [0.0027, 0.0086]	0.0058 [0.0027, 0.0084]	0.0043 [0.0028, 0.0071]	0.0029 [0.0027, 0.0056]
Random	0.0056 [0.0026, 0.0082]	0.0056 [0.0025, 0.0081]	0.0057 [0.0026, 0.0084]	0.0043 [0.0027, 0.0070]	0.0028 [0.0026, 0.0054]

The values between brackets represent the first and third quartiles.

Supplementary Table 3. Mean absolute error (MAE) values of the predicted MRI signal and the estimated quantitative parameters depending on the brain tissue type for synthetic data of the test subject with 500 selected measurements considering synthetic subject 3 as validation.

Test subject	Selection	All tissues	Grey matter	White matter
Signal MAE (a.u.)	CL+par	0.0590	0.0564	0.0529
	CL+eq	0.0588	0.0562	0.0527
	CL+NN	0.0590	0.0569	0.0533
	Uniform	0.0593	0.0566	0.0534
	Random	0.0589	0.0563	0.0528
$D_{\parallel}$ AE ($\mu m^2/ms$)	CL+par	**0.0479**	**0.0232**	**0.0207**
	CL+eq	0.0522	0.0286	0.0255
	CL+NN	0.0567	0.0291	0.0260
	Uniform	0.0712	0.0247	0.0230
	Random	0.0526	0.0292	0.0253
$D_{\perp}$ AE ($\mu m^2/ms$)	CL+par	**0.0342**	**0.0291**	**0.0245**
	CL+eq	0.0346	0.0332	0.0284
	CL+NN	0.0381	0.0350	0.0306
	Uniform	0.0356	0.0366	0.0317
	Random	0.0348	0.0321	0.0273
T_2^* AE (ms)	CL+par	110.6885	46.5543	36.7492
	CL+eq	**79.86271**	**34.7674**	**27.8943**
	CL+NN	125.6599	53.9051	42.6945
	Uniform	97.0816	41.9993	33.4727
	Random	91.2575	39.8236	31.8604
T_1 AE (ms)	CL+par	46.7066	22.9904	18.5973
	CL+eq	**37.8722**	**18.2376**	**14.9729**
	CL+NN	63.6459	28.4838	21.9788
	Uniform	40.4277	20.1224	16.6019
	Random	45.1126	23.5206	19.0837
S_0 AE (a.u.)	CL+par	0.0580	0.0432	0.0364
	CL+eq	**0.0442**	**0.0294**	0.0247
	CL+NN	0.0898	0.0636	0.0517
	Uniform	0.0539	0.0307	**0.0247**
	Random	0.0470	0.0362	0.0304
Eigenvector (1-dot product)	CL+par	0.0478	**0.0250**	0.0221
	CL+eq	0.0415	0.0259	**0.0206**
	CL+NN	0.0427	0.0296	0.0245
	Uniform	**0.0400**	0.0313	0.0287
	Random	0.0588	0.0317	0.0239

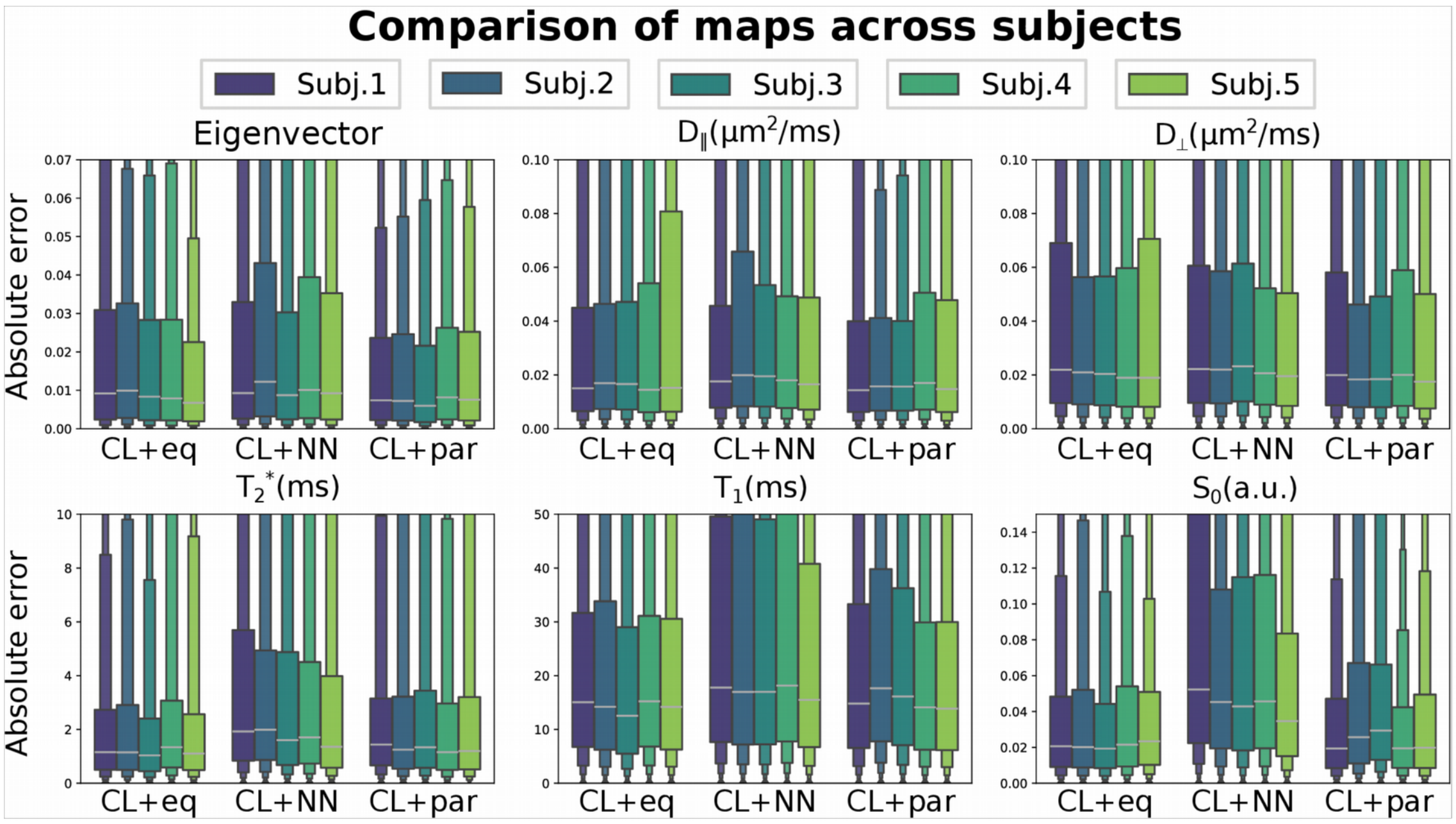

Supplementary Figure 6. Error of the estimated maps of the test synthetic subject for the diverse selection methods across validation subjects for the machine learning selection approaches for 500 selected submeasurements. The errors are shown for the whole brain. For the first eigenvector, the error was represented as one minus the dot product between the estimated and the ground truth maps to facilitate the interpretation. A.u. = arbitrary units.

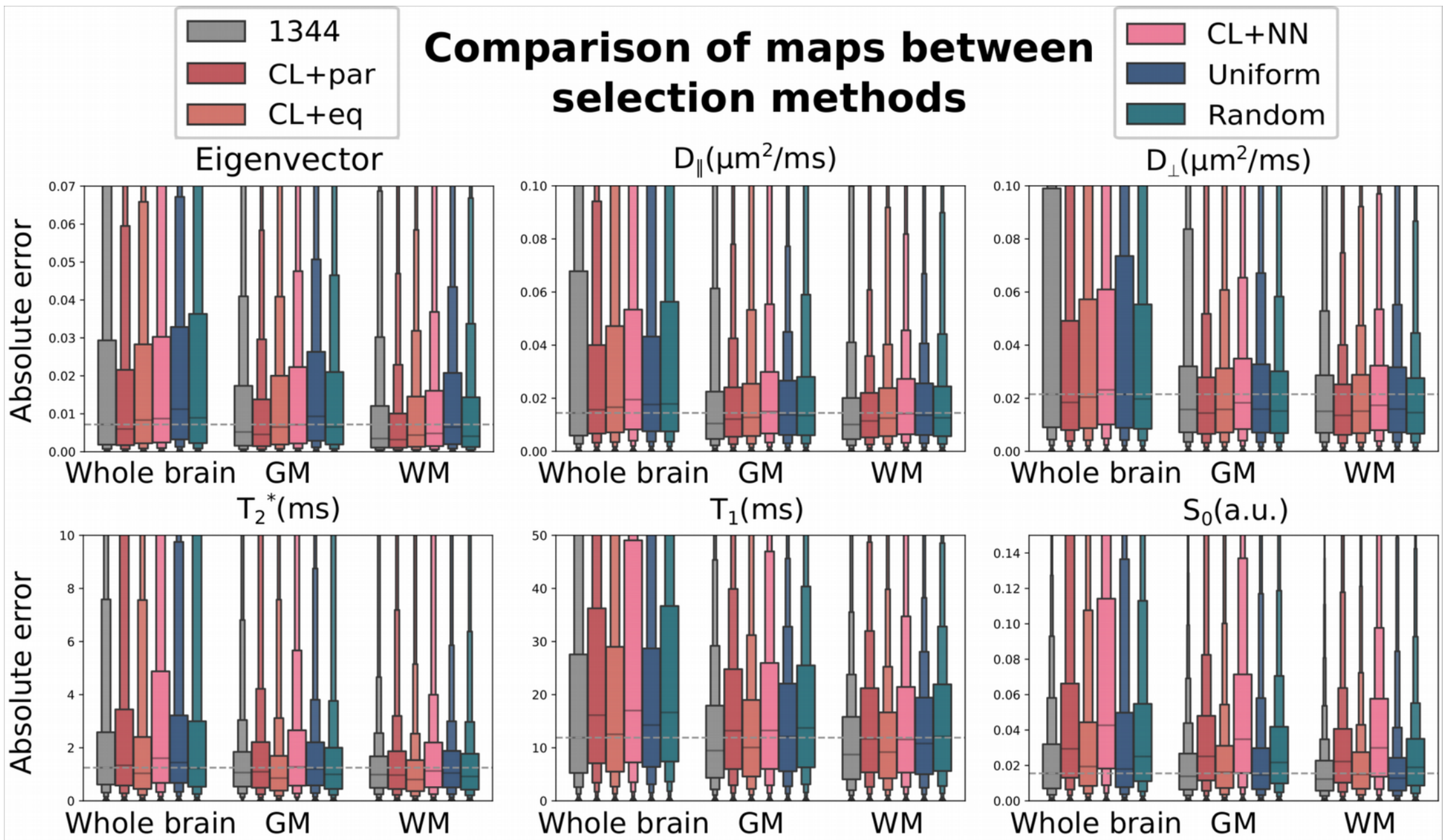

Supplementary Figure 7. Error of the estimated maps for the diverse selection methods for the test subject in one of the cross-validation procedures for 500 selected submeasurements. The boxplots show the absolute errors for the estimated parameters. The errors are shown for the whole brain, grey matter (GM) and white matter (WM). For the first eigenvector, the error was represented as one minus the dot product between the estimated and the ground truth maps to facilitate the interpretation. The boxplot *1344* represents the prediction of the whole dataset following the same estimation method as described for the subselected measurements. The dashed blue line shows the median error of the estimation on all 1344 measurements. The highest error have been omitted for visualization purposes. A.u. = arbitrary units.

Supplementary Table 4. Coefficient of variation (CV) of the error values of the estimated quantitative parameters for the synthetic test subject across validation subjects.

Selection (N=500)	$D_{\parallel}$	$D_{\perp}$	T_2^*	T_1	S_0	Eigenvector
CL+par	0.0830	0.1041	0.1406	0.0935	0.2154	0.1339
CL+eq	0.2726	0.1659	0.0853	**0.0532**	0.1020	0.1002
CL+NN	0.1168	0.0843	**0.0429**	0.1156	0.1805	0.1342
Uniform	0.0656	0.1097	0.1126	0.1039	0.0988	**0.0733**
Random	**0.0329**	**0.0427**	0.1064	0.1102	**0.0600**	0.1311

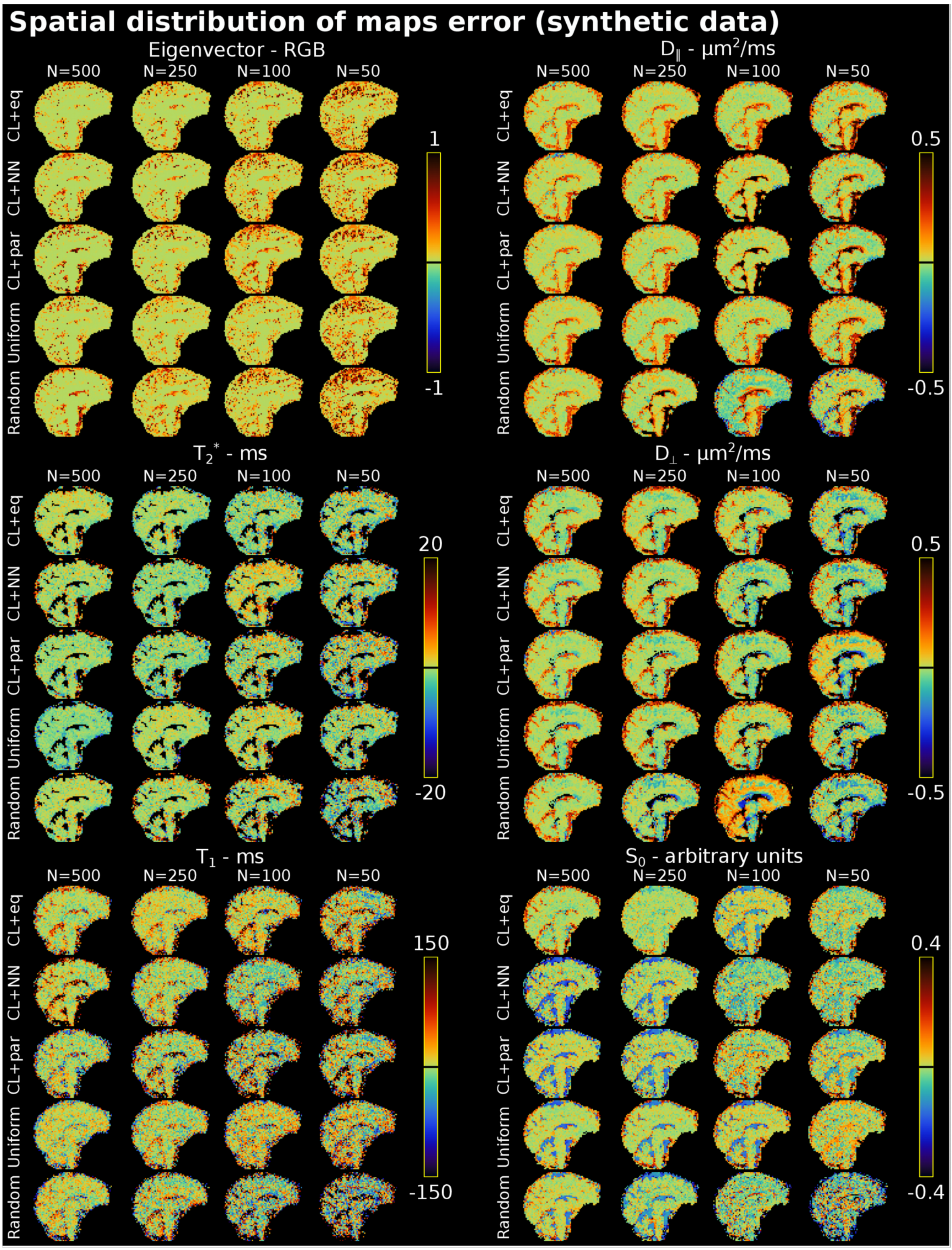

Supplementary Figure 8. Voxelwise error of the estimated maps from the synthetic test subject for five selection strategies and different number of subselected measurements considering subject 3 as validation. The black line in the colourbar represents the intermediate value, i.e., 0.5 for the eigenvector error and 0 for the remaining error values. RGB = Red Green Blue.

Supplementary Table 5. A) Coefficient of variation (CV) and interquartile range (IQR) of the mean absolute error (MAE) values of the predicted signal and the estimated quantitative parameters depending on the number of selected measurements from synthetic data of the test subject considering synthetic subject 3 as validation.

Test subject	Selection	N=500	N=250	N=100	N=50
CV					
Signal MAE	CL+par	0.5055	0.5050	0.5002	0.5019
	CL+eq	0.5052	0.4986	0.4985	0.4815
	CL+NN	0.5124	0.5040	0.4969	0.4825
	Uniform	**0.4967**	**0.4969**	0.4944	**0.4760**
	Random	0.5050	0.4970	**0.4821**	0.5080
$D_{\parallel}$ AE	CL+par	1.4689	1.5498	1.8393	1.5177
	CL+eq	1.4430	1.5557	1.4830	1.3481
	CL+NN	1.4486	1.6185	1.8339	1.5482
	Uniform	1.5236	**1.4783**	1.4479	1.4958
	Random	**1.4112**	1.7198	**1.2093**	**1.3211**
$D_{\perp}$ AE	CL+par	1.4776	1.5237	1.8290	1.7814
	CL+eq	**1.4212**	1.5571	1.4576	1.5753
	CL+NN	1.4325	1.5853	1.8122	1.7627
	Uniform	1.5798	**1.4856**	1.4389	1.6173
	Random	1.4723	1.5547	**1.2848**	**1.5032**
T_2^* AE	CL+par	3.3102	3.3676	3.2375	3.2082
	CL+eq	3.3483	3.2552	3.2750	3.1860
	CL+NN	**3.2550**	3.2862	3.3240	3.3078
	Uniform	3.3120	3.3282	3.2403	4.5758
	Random	3.3311	**3.2387**	**3.1474**	**3.0370**
T_1 AE	CL+par	2.2807	**2.0213**	**1.9177**	2.3461
	CL+eq	2.4345	2.1396	2.0715	2.2627
	CL+NN	**2.0395**	2.2663	2.0862	2.1675
	Uniform	2.8227	2.0303	2.3578	**1.9140**
	Random	2.1876	2.0950	1.9898	1.9590
S_0 AE	CL+par	**1.2272**	1.1575	1.1924	1.1731
	CL+eq	1.4524	**1.1176**	**1.0516**	1.1878
	CL+NN	1.3170	1.1303	1.2107	1.2751
	Uniform	1.9781	1.3986	1.1630	**1.1430**
	Random	1.2394	1.3520	1.6995	1.4655
Eigenvector AE	CL+par	2.9195	2.4609	**1.7398**	1.4761
	CL+eq	2.6922	2.4317	2.1786	**1.3549**
	CL+NN	2.5233	2.5766	1.8393	1.3898
	Uniform	2.4788	2.1123	2.0271	1.3702
	Random	**2.2867**	**1.7658**	1.9614	1.4635
IQR					
Signal MAE (a.u.)	CL+par	0.0312 [0.0404, 0.0717]	0.0317 [0.0410, 0.0727]	0.0320 [0.0423, 0.0742]	0.0342 [0.0436, 0.0778]
	CL+eq	0.0310 [0.0404, 0.0714]	0.0311 [0.0408, 0.0719]	0.0314 [0.0416, 0.0730]	0.0331 [0.0436, 0.0767]

	CL+NN	0.0317 [0.0407, 0.0724]	0.0313 [0.0406, 0.0720]	0.0317 [0.0421, 0.0738]	0.0326 [0.0432, 0.0758]
	Uniform	0.0313 [0.0409, 0.0722]	0.0310 [0.0411, 0.0721]	0.0315 [0.0416, 0.0731]	0.0335 [0.0438, 0.0773]
	Random	0.0312 [0.0404, 0.0716]	0.0320 [0.0422, 0.0742]	0.0372 [0.0446, 0.0818]	0.0339 [0.0458, 0.0856]
$D_{\parallel}$ AE ($\mu m^2/ms$)	CL+par	**0.0333** [0.0067, 0.0400]	**0.0364** [0.0077, 0.0441]	0.0786 [0.0103, 0.0889]	0.0891 [0.0150, 0.1041]
	CL+eq	0.0400 [0.0071, 0.0472]	0.0397 [0.0080, 0.0477]	**0.0443** [0.0102, 0.0545]	0.0802 [0.0151, 0.0953]
	CL+NN	0.0451 [0.0083, 0.0534]	0.0381 [0.0076, 0.0457]	0.0727 [0.0113, 0.0841]	0.0725 [0.0142, 0.0867]
	Uniform	0.0357 [0.0076, 0.0432]	0.0414 [0.0085, 0.0499]	0.0453 [0.0110, 0.0563]	**0.0708** [0.0160, 0.0867]
	Random	0.0488 [0.0075, 0.0563]	0.0590 [0.0102, 0.0692]	0.0690 [0.0244, 0.0934]	0.1011 [0.0203, 0.1214]
$D_{\perp}$ AE ($\mu m^2/ms$)	CL+par	**0.0411** [0.0080, 0.0491]	0.0517 [0.0096, 0.0613]	0.0678 [0.0121, 0.0799]	0.0865 [0.0242, 0.1107]
	CL+eq	0.0479 [0.0087, 0.0566]	0.0550 [0.0101, 0.0652]	0.0608 [0.0129, 0.0737]	**0.0779** [0.0191, 0.0970]
	CL+NN	0.0512 [0.0102, 0.0614]	**0.0484** [0.0095, 0.0579]	0.0694 [0.0136, 0.0829]	0.0905 [0.0198, 0.1103]
	Uniform	0.0652 [0.0089, 0.0740]	0.0572 [0.0111, 0.0683]	**0.0584** [0.0159, 0.0742]	0.0804 [0.0203, 0.1007]
	Random	0.0466 [0.0085, 0.0551]	0.0639 [0.0127, 0.0765]	0.1202 [0.0457, 0.1659]	0.1112 [0.0249, 0.1362]
T_2^* AE (ms)	CL+par	2.8674 [0.5763, 3.4437]	3.9478 [0.8792, 4.8271]	4.7579 [1.1396, 5.8975]	6.5933 [1.6191, 8.2124]
	CL+eq	**1.9562** [0.4485, 2.4047]	2.2879 [0.5367, 2.8245]	3.4859 [0.8788, 4.3647]	**4.6367** [1.1435, 5.7802]
	CL+NN	4.2014 [0.6762, 4.8775]	3.0654 [0.7348, 3.8002]	4.1632 [0.9048, 5.0680]	5.1412 [1.1711, 6.3123]
	Uniform	2.5418 [0.6759,	**2.2209** [0.5026,	**3.4199** [0.7343,	4.7483 [1.0488,

		3.2177]	2.7234]	4.1542]	5.7971]
	Random	2.4664 [0.5307, 2.9971]	4.3283 [0.7646, 5.0929]	7.6788 [1.2593, 8.9382]	13.5129 [1.9674, 15.4803]
T_1 AE (ms)	CL+par	29.1783 [7.0717, 36.2501]	42.5610 [9.1971, 51.7581]	62.4395 [14.7575, 77.1970]	55.1890 [13.2292, 68.4182]
	CL+eq	23.5121 [5.4913, 29.0034]	**22.4724** [6.2063, 28.6787]	38.2740 [8.8776, 47.1516]	52.2250 [12.8390, 65.0640]
	CL+NN	41.8149 [7.2338, 49.0488]	25.8469 [6.7910, 32.6379]	41.7437 [11.1089, 52.8527]	54.1133 [13.0996, 67.2130]
	Uniform	**22.3065** [6.3915, 28.6981]	26.1998 [7.5142, 33.7140]	**35.7508** [10.4330, 46.1838]	**50.9285** [14.1719, 65.1004]
	Random	29.2803 [7.4009, 36.6812]	42.3922 [10.6745, 53.0666]	65.3411 [16.0772, 81.4183]	101.2334 [24.0292, 125.2626]
S_0 AE (a.u.)	CL+par	0.0328 [0.0130, 0.0662]	0.0604 [0.0152, 0.0757]	0.0659 [0.0196, 0.0855]	0.0701 [0.0173, 0.0875]
	CL+eq	0.0358 [0.0084, 0.0442]	**0.0282** [0.0088, 0.0371]	**0.0572** [0.0164, 0.0736]	**0.0506** [0.0170, 0.0677]
	CL+NN	0.0473 [0.0183, 0.1149]	0.0484 [0.0135, 0.0619]	0.0644 [0.0194, 0.0838]	0.0617 [0.0209, 0.0826]
	Uniform	**0.0213** [0.0077, 0.0492]	0.0443 [0.0091, 0.0534]	0.0577 [0.0137, 0.0715]	0.0562 [0.0189, 0.0750]
	Random	0.0291 [0.0111, 0.0547]	0.0833 [0.0167, 0.1000]	0.0890 [0.0240, 0.1130]	0.1495 [0.0432, 0.1927]
Eigenvector AE (1-dot product)	CL+par	**0.0199** [0.0017, 0.0216]	0.0321 [0.0032, 0.0353]	0.0872 [0.0062, 0.0934]	**0.0688** [0.0093, 0.0781]
	CL+eq	0.0261 [0.0022, 0.0283]	0.0314 [0.0036, 0.0351]	**0.0414** [0.0057, 0.0471]	0.0926 [0.0123, 0.1049]
	CL+NN	0.0279 [0.0024, 0.0303]	**0.0264** [0.0027, 0.0291]	0.0688 [0.0076, 0.0764]	0.0886 [0.0120, 0.1006]
	Uniform	0.0296 [0.0032, 0.0329]	0.0429 [0.0047, 0.0476]	0.0490 [0.0061, 0.0552]	0.0873 [0.0115, 0.0989]
	Random	0.0341 [0.0023, 0.0363]	0.0643 [0.0083, 0.0725]	0.0613 [0.0079, 0.0692]	0.1518 [0.0141, 0.1659]

The values between brackets represent the first and third quartiles.

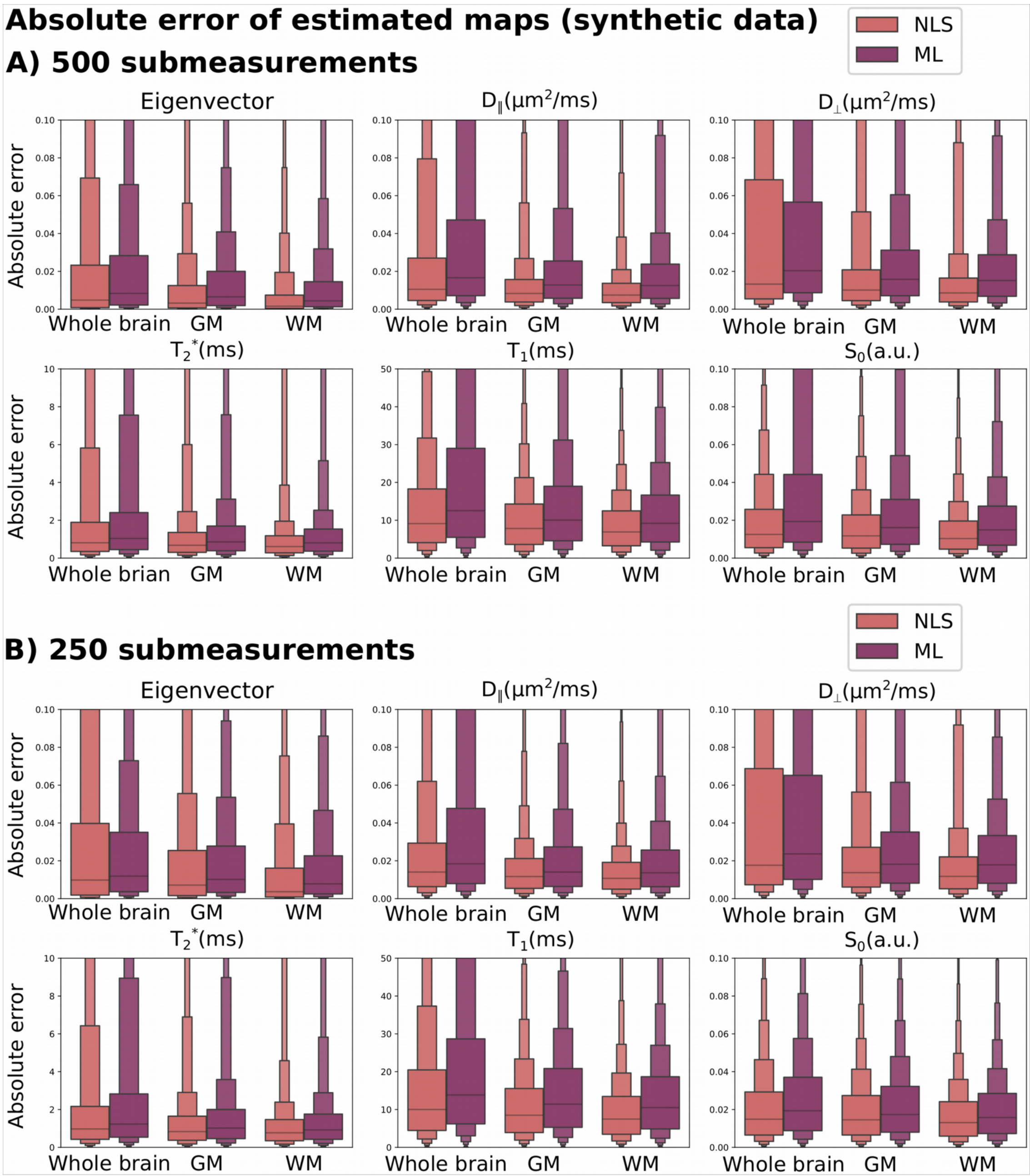
Absolute error of estimated maps (synthetic data)
A) 500 submeasurements
NLS
ML
Eigenvector
D∥(μm²/ms)
D⊥(μm²/ms)
T2*(ms)
T1(ms)
S0(a.u.)
Absolute error
Whole brain
Whole brian
GM
WM
B) 250 submeasurements
NLS
ML

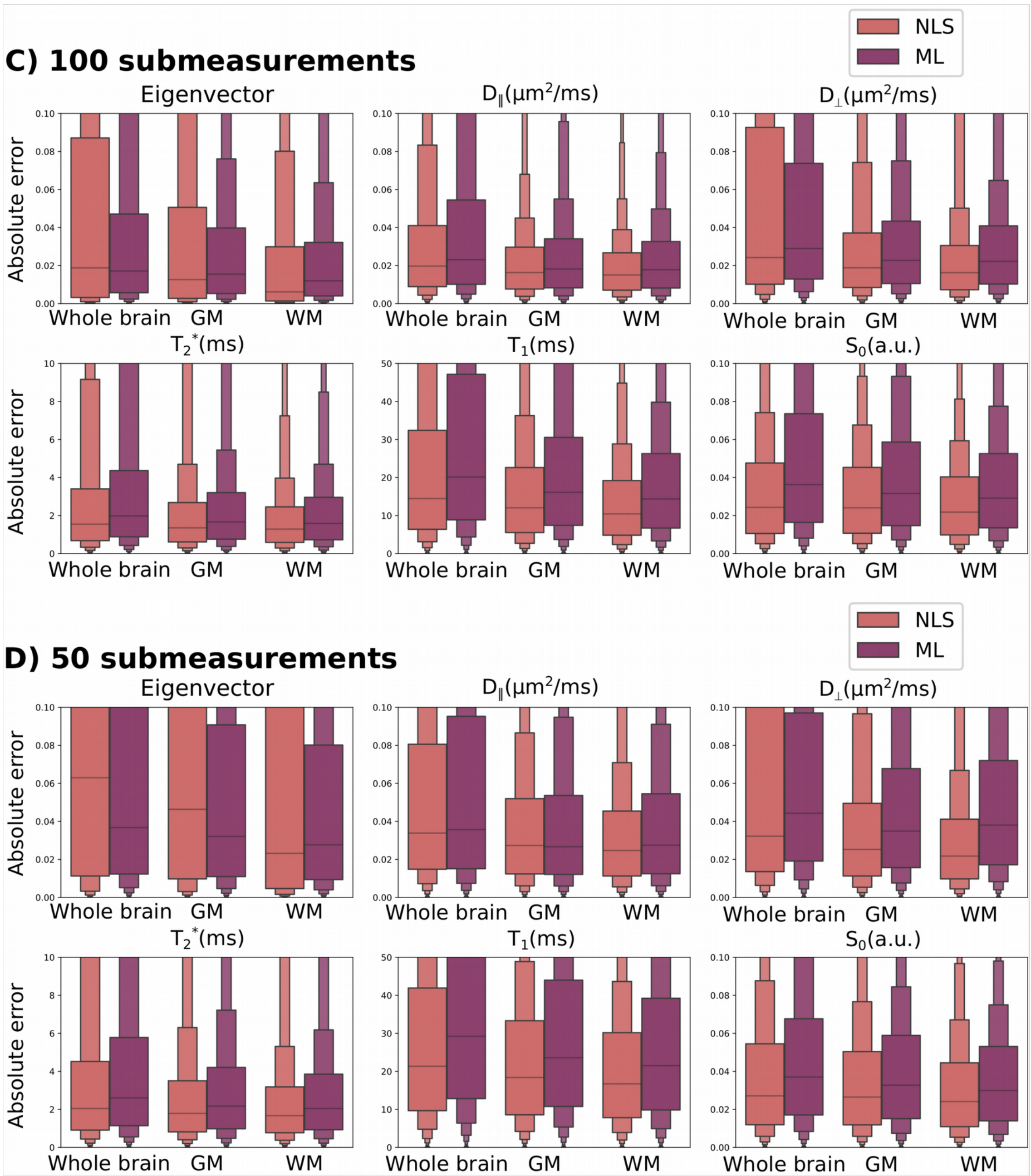

Supplementary Figure 9. Comparison of the estimation of synthetic quantitative volumes from the test subject depending on different estimation strategies using machine learning (ML) and non-linear squares (NLS) approaches for: A) 500 selected measurements; B) 250 selected measurements; C) 100 selected measurements; D) 50 selected measurements. The absolute error of the quantitative map is shown for the whole brain, grey matter (GM) and white matter (WM) using the measurements from the CL+eq approach. Synthetic subject 3 was considered as validation.

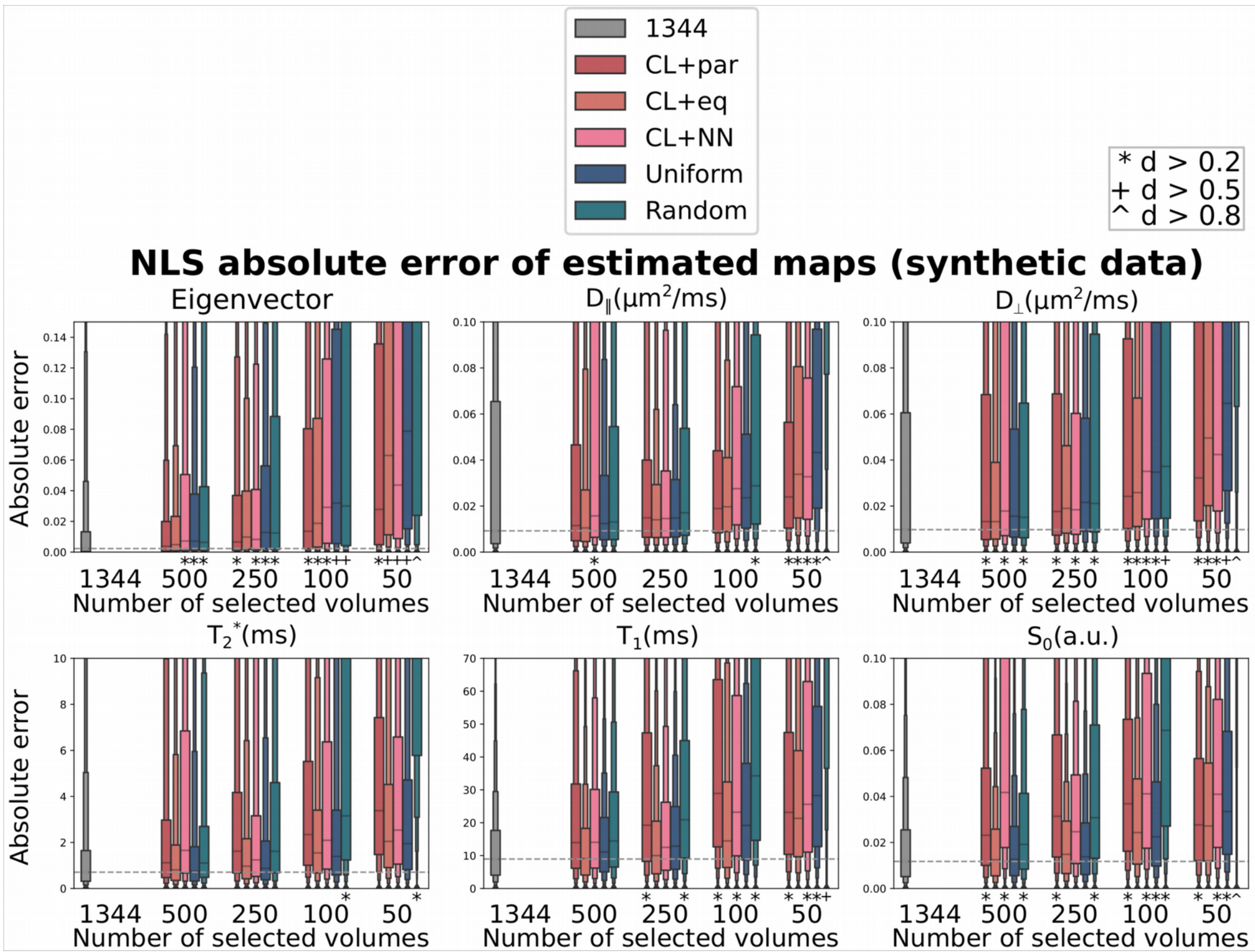

Supplementary Figure 10. Comparison of the non-linear squares (NLS) estimation of synthetic quantitative volumes from the test subject depending on the number of subselected measurements for each selection approach and using subject 3 as validation. The boxplot *1344* represents the prediction of the whole dataset via NLS using all measurements, i.e., the difference between the ground truth and the fitting performed using all the 1344 measurements. The dashed blue line shows the median error of the 1344 estimation. The highest errors have been omitted for visual purpose. A.u. = arbitrary units. *,+;^ = small/medium/large effect size (d > 0.2; 0.5; 0.8) of the difference of the errors of the estimated parameters between the estimation from the selected subprotocol and the estimation with the whole dataset.

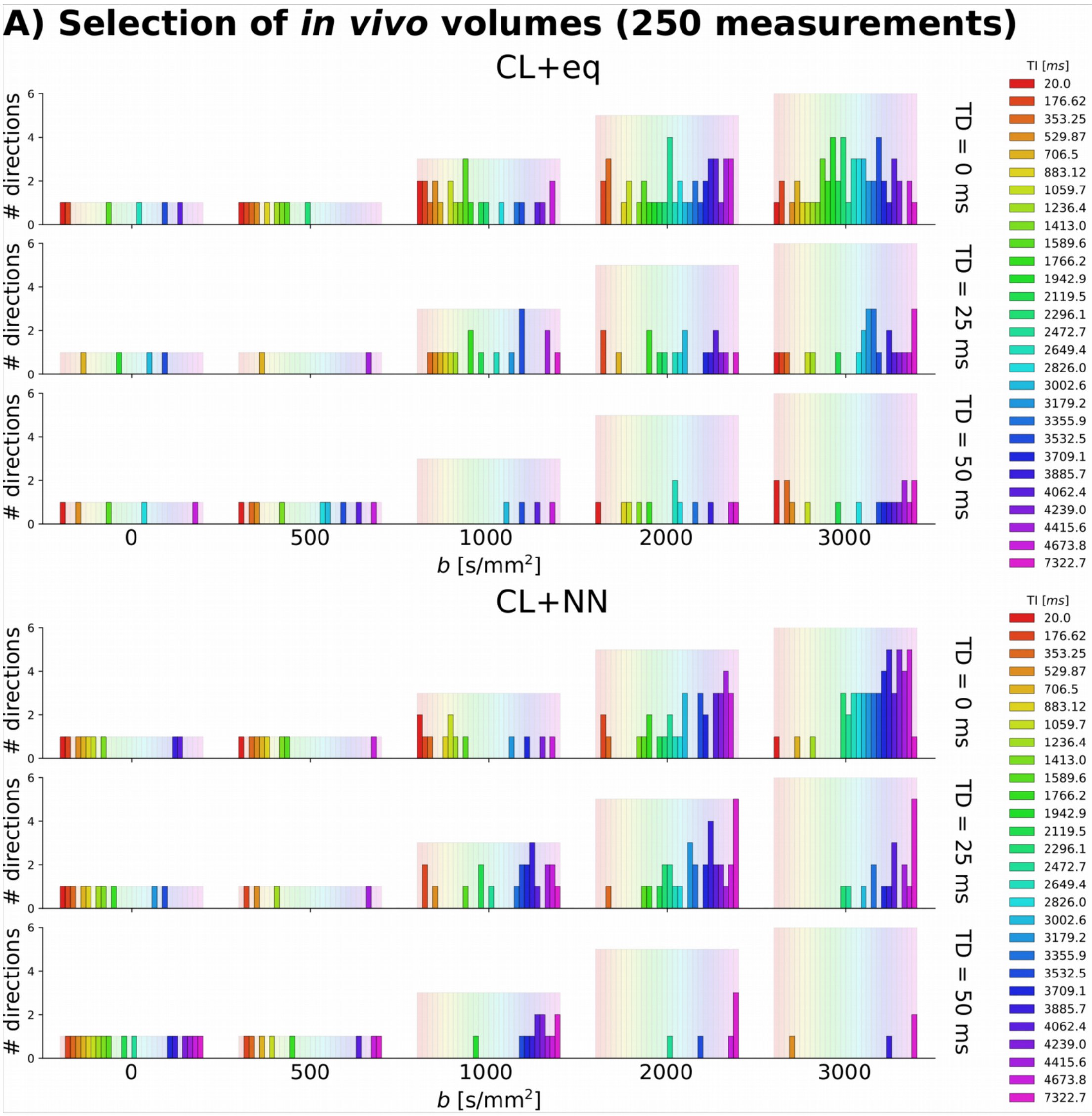
A) Selection of in vivo volumes (250 measurements)
CL+eq
CL+NN
directions
b [s/mm2]
0
500
1000
2000
3000
TD = 0 ms
TD = 25 ms
TD = 50 ms
TI [ms]
20.0
176.62
353.25
529.87
706.5
883.12
1059.7
1236.4
1413.0
1589.6
1766.2
1942.9
2119.5
2296.1
2472.7
2649.4
2826.0
3002.6
3179.2
3355.9
3532.5
3709.1
3885.7
4062.4
4239.0
4415.6
4673.8
7322.7

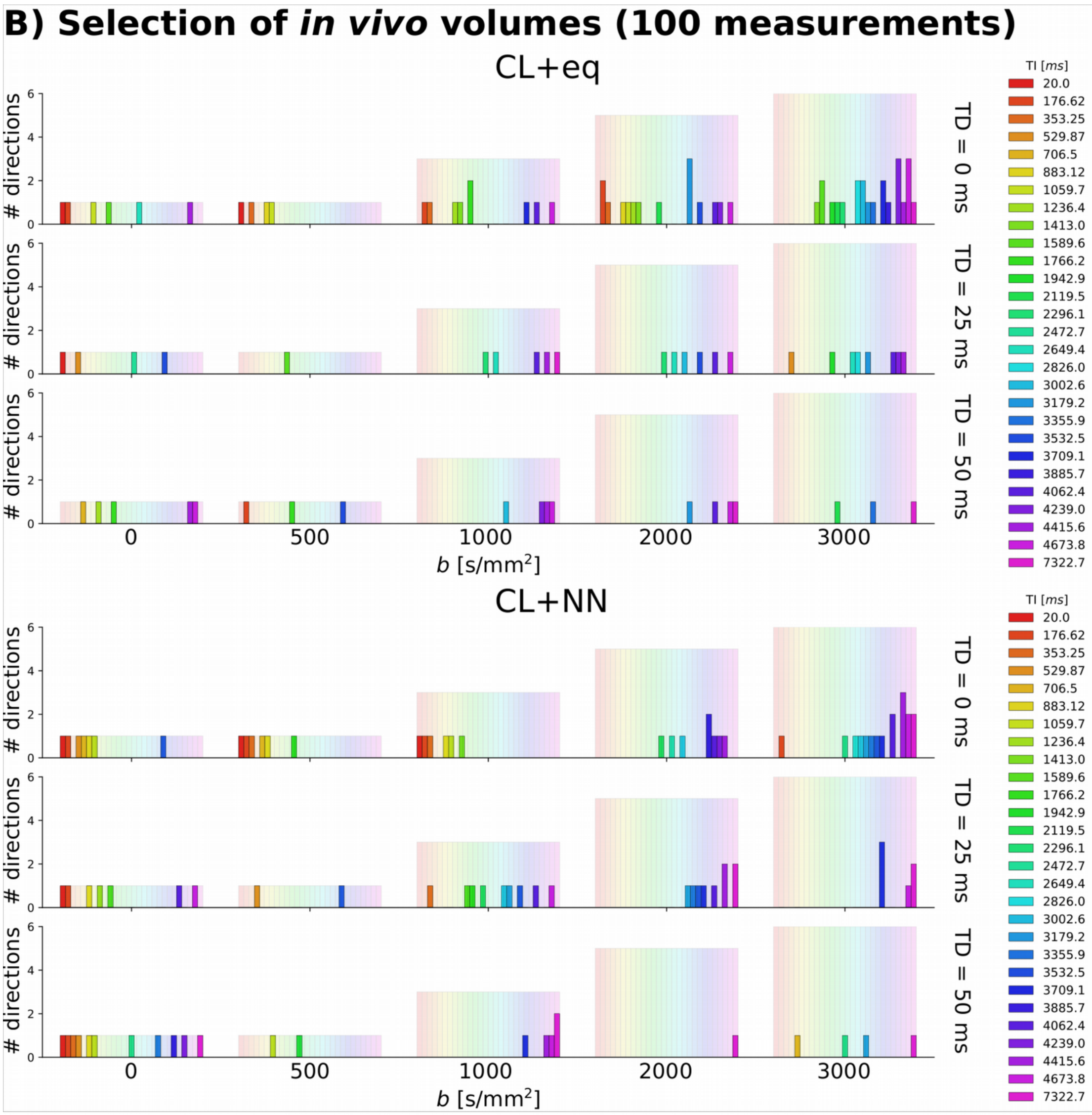
B) Selection of *in vivo* volumes (100 measurements)
CL+eq
CL+NN
# directions
TD = 0 ms
TD = 25 ms
TD = 50 ms
0
500
1000
2000
3000
b [s/mm²]
TI [ms]
20.0
176.62
353.25
529.87
706.5
883.12
1059.7
1236.4
1413.0
1589.6
1766.2
1942.9
2119.5
2296.1
2472.7
2649.4
2826.0
3002.6
3179.2
3355.9
3532.5
3709.1
3885.7
4062.4
4239.0
4415.6
4673.8
7322.7

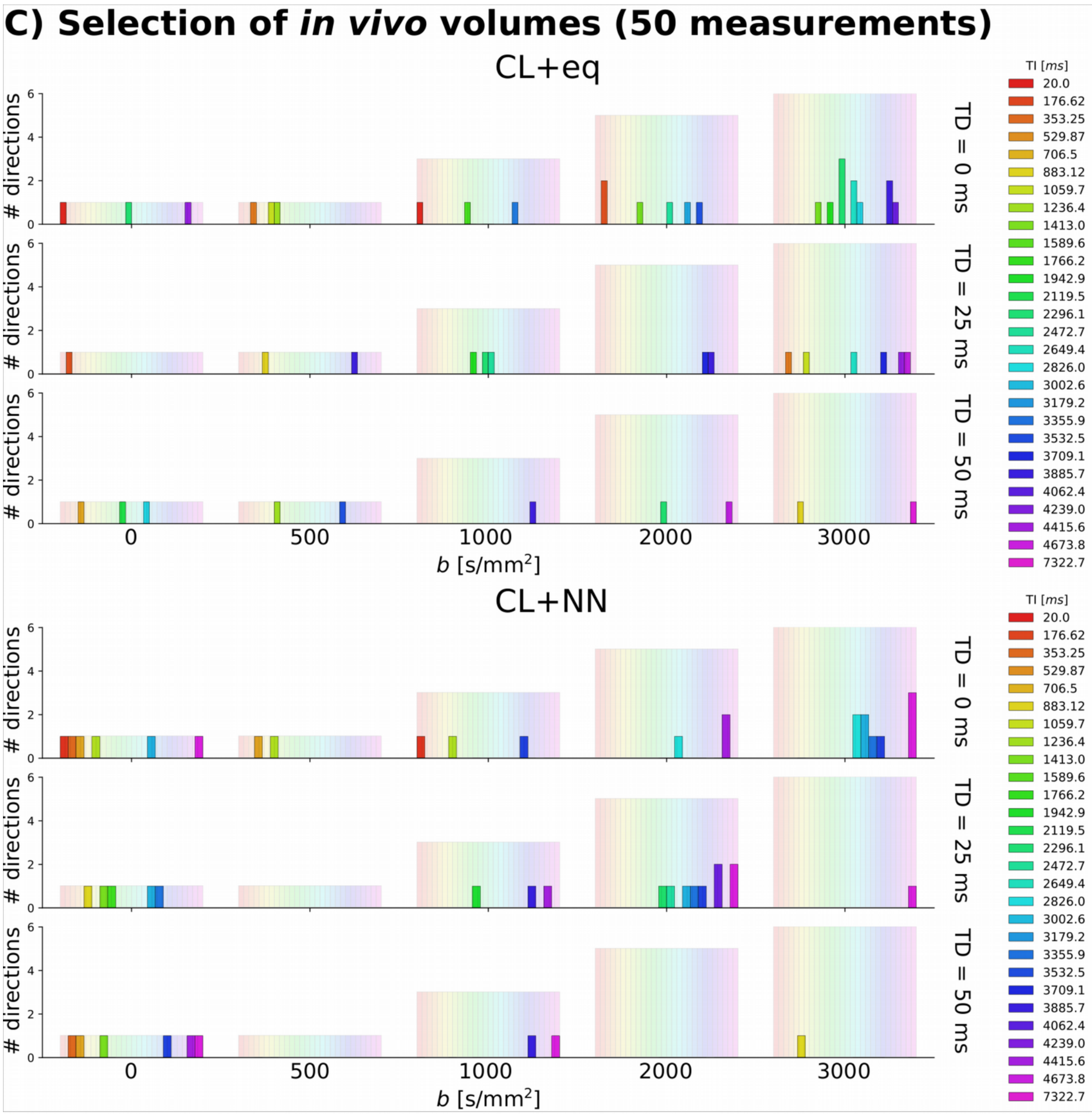

Supplementary Figure 11. Comparison of the selected 250 (A), 100 (B) and 50 measurements (C) between the machine learning methods in *in vivo* data. The histograms represent the mean number of chosen volumes (gradient orientations) for each b-value (0, 500, 1000, 2000 and 3000 s/mm²), TD (0, 25 and 50 ms), and TI (28 values from 20 to 7322.7 ms) for one validation subject.

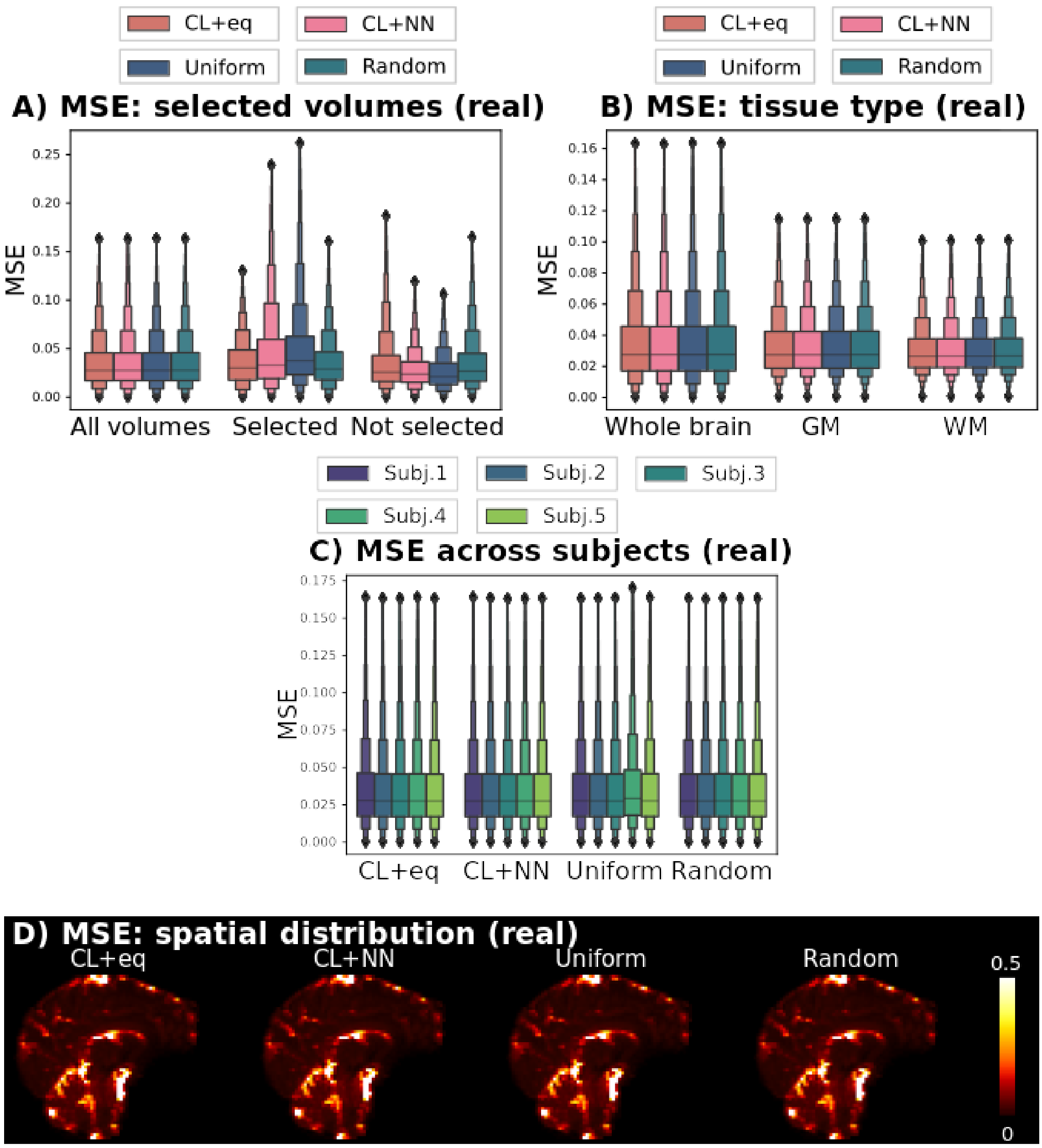

Supplementary Figure 12. Mean squared error (MSE) of the predicted *in vivo* measurements from the test subject for four selection strategies and 500 selected measurements subdivided by: A) MRI volumes (whole dataset, and volumes used and unused to train the estimation network); B) Tissue type (whole brain, grey matter-GM, and white matter-WM). C) All cross-validation subjects for 500 subselected measurements organised by selection method. D) Spatial distribution of the MSE for all volumes and 500 subselected measurements considering subject 3 as validation.

Supplementary Table 6. Mean squared error (MSE) values of the predicted signal on the test subject depending on the number of selected measurements for *in vivo* data using subject 3 as validation.

	Selection	N=500	N=250	N=100	N=50
Signal MSE (a.u.)	CL+eq	0.0519	0.0518	0.0537	0.0538
	CL+NN	0.0518	0.0519	0.0569	0.0579
	Uniform	0.0518	0.0523	0.0620	0.0536
	Random	0.0519	0.0533	0.0604	0.0609

Supplementary Table 7. Mean squared error (MSE) values of the predicted signal on the test subject depending on the validation subject for *in vivo* data.

	Selection	Subject 1	Subject 2	Subject 3	Subject 4	Subject 5
Signal MSE (a.u.)	CL+eq	0.0523	0.0518	0.0519	0.0519	0.0518
	CL+NN	0.0519	0.0519	0.0518	0.0519	0.0518
	Uniform	0.0520	0.0518	0.0518	0.0544	0.0520
	Random	0.0519	0.0519	0.0519	0.0518	0.0518

Supplementary Table 8. Coefficient of variation (CV) and interquartile range (IQR) of the MSE of the predicted signal depending on the number of selected measurements from *in vivo* data of the test subject considering subject 3 as validation.

	Selection	N=500	N=250	N=100	N=50
CV					
Signal MSE (a.u.)	CL+eq	1.8646	1.8099	1.8416	1.8398
	CL+NN	**1.8267**	1.8698	1.8372	1.7953
	Uniform	1.8317	1.8549	1.8195	1.7757
	Random	1.8411	**1.7669**	**1.7386**	**1.7281**
IQR					
Signal MSE (a.u.)	CL+eq	0.0334 [0.0176, 0.0510]	0.0334 [0.0177, 0.0511]	**0.0337** [0.0178, 0.0515]	**0.0348** [0.0184, 0.0532]
	CL+NN	**0.0332** [0.0177, 0.0508]	**0.0333** [0.0177, 0.0510]	0.0362 [0.0186, 0.0547]	0.0352 [0.0183, 0.0536]
	Uniform	0.0333 [0.0176, 0.0509]	0.0335 [0.0179, 0.0514]	0.0365 [0.0184, 0.0549]	0.0348 [0.0184, 0.0532]
	Random	0.0334 [0.0177, 0.0511]	0.0349 [0.0182, 0.0531]	0.0409 [0.0201, 0.0610]	0.0402 [0.0205, 0.0608]

The values between brackets represent the first and third quartiles

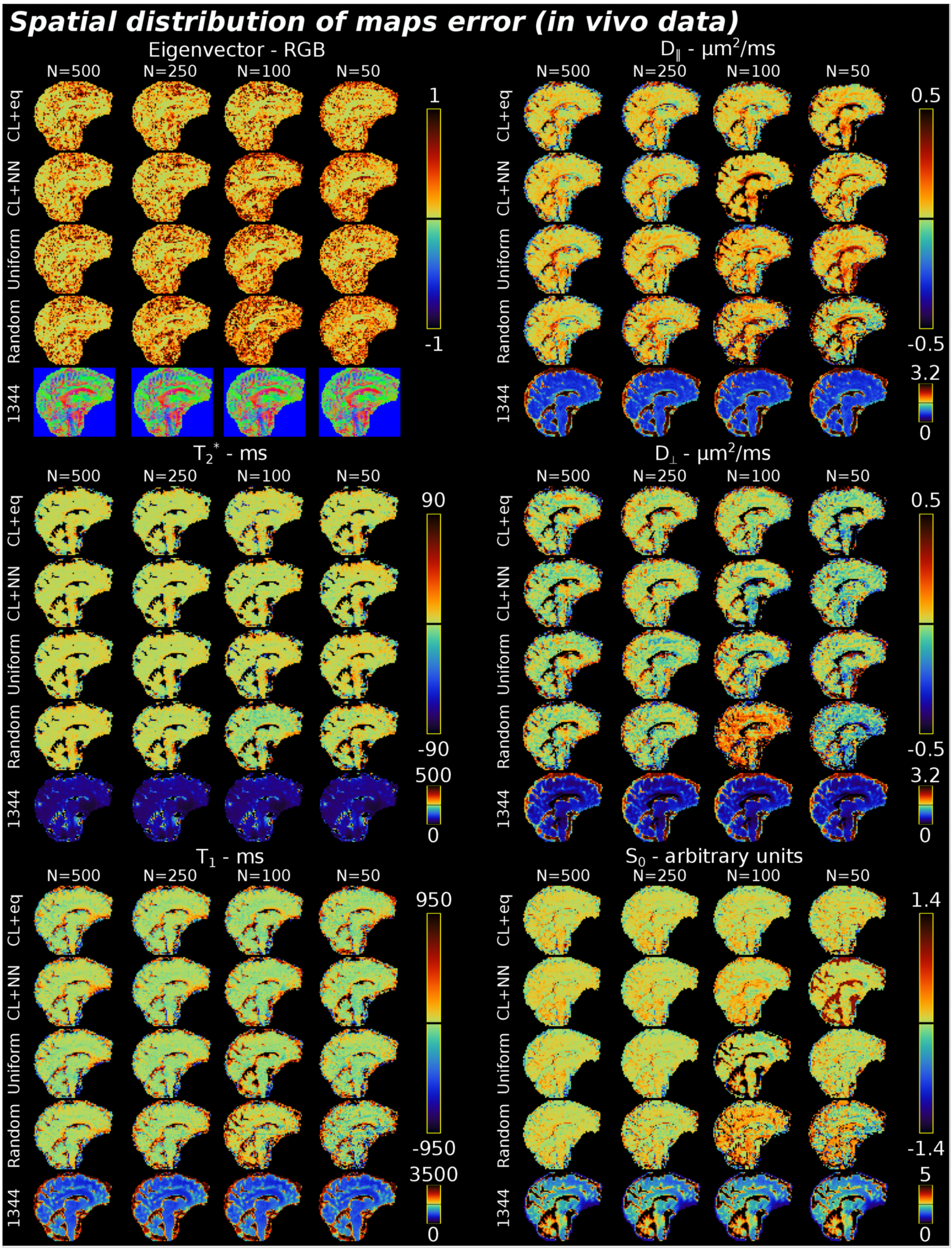

Supplementary Figure 13. Voxelwise error compared to the estimation from the whole dataset of the estimated maps from the test subject considering subject 3 as validation for four selection strategies and different number of subselected measurements (*N*). The eigenvector error was represented as one minus the dot product between the estimated and reference maps. The black line in the colourbar represents the intermediate value, i.e., 0.5 for the eigenvector error and 0 for the remaining error values (the same for the reference maps). For each map, the bottom row represents the estimation from the whole dataset. RGB = Red Green Blue.
